# Identification of Tomato Infecting Viruses That Co-Isolate with Nanovesicles Using a Combined Proteomics and Electron-Microscopic Approach

**DOI:** 10.3390/nano11081922

**Published:** 2021-07-26

**Authors:** Ramila Mammadova, Immacolata Fiume, Ramesh Bokka, Veronika Kralj-Iglič, Darja Božič, Matic Kisovec, Marjetka Podobnik, Apolonija Bedina Zavec, Matej Hočevar, Gabriella Gellén, Gitta Schlosser, Gabriella Pocsfalvi

**Affiliations:** 1 Extracellular Vesicles and Mass Spectrometry Laboratory, Institute of Biosciences and BioResources, National Research Council of Italy, 80131 Naples, Italy; ramila.mammadova797@gmail.com (R.M.); immacolata.fiume@ibbr.cnr.it (I.F.); ramesh.chem2008@gmail.com (R.B.); 2 Laboratory of Clinical Biophysics, Faculty of Health Sciences, University of Ljubljana, SI-1000 Ljubljana, Slovenia; veronika.kralj-iglic@fe.uni-lj.si (V.K.-I.); darja.bozic@fe.uni-lj.si (D.B.); 3 Department of Molecular Biology and Nanobiotechnology, National Institute of Chemistry, SI-1000 Ljubljana, Slovenia; matic.kisovec@ki.si (M.K.); marjetka.podobnik@ki.si (M.P.); polona.bedina@ki.si (A.B.Z.); 4 Institute of Metals and Technology, SI-1000 Ljubljana, Slovenia; matej.hocevar@imt.si; 5 MTA-ELTE Lendület Ion Mobility Mass Spectrometry Research Group, ELTE Eötvös Loránd University, Institute of Chemistry, H-1117 Budapest, Hungary; gabgellen@staff.elte.hu (G.G.); gitta.schlosser@ttk.elte.hu (G.S.)

**Keywords:** tomato, plant viruses, tomato brown rugose fruit virus, tomato mosaic virus, tomato spotted wilt virus, extracellular vesicles, capsid protein

## Abstract

Plant-derived nanovesicles (NVs) have attracted interest due to their anti-inflammatory, anticancer and antioxidative properties and their efficient uptake by human intestinal epithelial cells. Previously we showed that tomato (*Solanum lycopersicum* L.) fruit is one of the interesting plant resources from which NVs can be obtained at a high yield. In the course of the isolation of NVs from different batches of tomatoes, using the established differential ultracentrifugation or size-exclusion chromatography methods, we occasionally observed the co-isolation of viral particles. Density gradient ultracentrifugation (gUC), using sucrose or iodixanol gradient materials, turned out to be efficient in the separation of NVs from the viral particles. We applied cryogenic transmission electron microscopy (cryo-TEM), scanning electron microscopy (SEM) for the morphological assessment and LC–MS/MS-based proteomics for the protein identification of the gradient fractions. Cryo-TEM showed that a low-density gUC fraction was enriched in membrane-enclosed NVs, while the high-density fractions were rich in rod-shaped objects. Mass spectrometry–based proteomic analysis identified capsid proteins of tomato brown rugose fruit virus, tomato mosaic virus and tomato mottle mosaic virus. In another batch of tomatoes, we isolated tomato spotted wilt virus, potato virus Y and southern tomato virus in the vesicle sample. Our results show the frequent co-isolation of plant viruses with NVs and the utility of the combination of cryo-TEM, SEM and proteomics in the detection of possible viral contamination.

## 1. Introduction

Cell-derived submicron particles isolated from plant resources [1,2,3,4,5,6,7] are gaining attention both as complex biomaterials with health-promoting effects [8,9] and as delivery vectors for exogenous substances [10]. Nanometer-sized vesicles (NVs) have been isolated from many different plants, such as ginger [6,7,11], carrots [12], citrus species [4,13], grapes [1,2], tomato [1,14], blueberry [1], coconut [1], broccoli [15], wheat [16], etc., and even dried plant material [5]. Several of these isolates were shown to have anticancer [3,17,18], anti-inflammatory [7,11,15] or anti-senescence [19] activities in vitro or in vivo. For example, NVs derived from *Panax Ginseng* root can inhibit melanoma cell growth through macrophage polarization [18]. NVs isolated from grapefruit [20] and citrus [3] fruit juice were shown to exhibit anticancer activities in human melanoma and other cell lines. Numerous studies demonstrated the potential of plant-derived vesicles for clinical [9,21], pharmaceutical [9] and cosmeceutical [22] applications. For example, native NVs from ginger root have been shown to be efficient in the treatment of alcohol-induced liver damage in vivo [11]. Both broccoli- and grapefruit-derived nanoparticles prevent dextran sulfate sodium–induced colitis in murine models [15,23]. NVs isolated from wheat grass juice exert activity in the wound-healing process based on in vitro testing [16] and ginseng-derived vesicles exhibited anti-senescence effects on human skin cells [19]. Besides the biological activities, plant-derived NVs are used as carriers for the delivery of exogenous molecules/drug cargo. For instance, grapefruit-juice-derived NVs ameliorated the delivery of exogenous proteins to human cells compared to the same proteins without vesicles [10]. Moreover, the administration of ginger-exosome-like NVs loaded with exogenous therapeutic RNA efficiently inhibited tumor growth in a mice model [10].

A recent work shows that NVs can be obtained with a high yield from tomatoes, using a combination of differential centrifugation (dUC) and size-exclusion chromatography (SEC) methods [14]. Tomato (*Solanum lycopersicum* L.) is one of the leading vegetable crops worldwide. Tomatoes are consumed fresh, as well as cooked, or can be processed into commercially available stabilized processed products, such as canned tomatoes, pastes, purees and ketchups. Tomato fruit contains various bioactive compounds and minerals, such as carotenoids, as well as lycopene associated with the numerous health benefits of tomatoes, including anti-inflammatory and anticancer activities; and reduced risk of cardiovascular diseases, diabetes and obesity [24,25].

Tomato plants are continuously confronted by various viral, bacterial, fungal and pest pathogens. Over recent years, several viral diseases have emerged affecting the quality of the fruit and contributing to the pre- and post-harvest losses both in field and greenhouse tomato. Emerging viruses spread very fast and require prompt intervention for their control and prevention [26]. Most of the viral diseases in tomato are attributed to single-stranded RNA (ssRNA) viruses [26]. Tomato mosaic virus (ToMV), for example, is a positive-sense ssRNA virus from the genus *Tobamovirus* [27]. Many commercial tomato varieties contain dominant resistance genes for ToMV. The capsid protein, also called coat protein (CP), of ToMV self-assembles to form rod-shaped virions about 18 nm in diameter with a central canal enclosing the viral genomic RNA. Tomato mottle mosaic virus (ToMMV) is another representative of *Tobamoviruses* that infects tomato plants worldwide [28]. ToMMV is a rigid rod-shaped particle that is about 300 nm long [29]. Another well-known, highly contagious *Tobamovirus* from the family *Virgaviridae* is the tomato brown rugose fruit virus (ToBRFV). ToBRFV was first identified in Jordan and Israel, but since then, its presence has been reported in many different countries in Europe, Asia and North America [30]. ToBRFV is a monopartite positive-sense ssRNA virus that is 300 nm long and 17 nm in diameter. Its genome contains four open reading frames (ORFs) [31]. Two ORFs (ORF1a and ORF1b) encode the replication-related proteins, ORF2 encodes a movement protein (MP) and ORF3 encodes the CP [32]. ToBRFV is easily transmitted by mechanical contact and via infected seeds. Typically, the symptoms caused by ToBRFV infection are yellow spots or brown wrinkled patches on the infected fruits but sometimes deformation and necrosis. Another virus that causes severe disease of tomato is the tomato spotted wilt virus (TSWV), *Orthotospovirus* genus, *Tospoviridae* family [33,34]. Transmission of TSWV mainly occurs by thrips, which also act as vectors of the virus [34]. TSWV has a tripartite ssRNA genome of negative/ambisense polarity, consisting of the large, medium and small RNA segments [35] that encode for a 331.5 kDa protein, nonstructural polyproteins and a viral nucleocapsid N protein [34,35]. The two envelope membrane glycoproteins (Gn and Gc) play a crucial role in TSWV acquisition by the vector thrips [35]. TSWV has a roughly spherical shape with a diameter between 80 and 120 nm [34]. The symptoms of the plants infected by TSWV are small dark-brown spots on leaves, stems and petioles; light green rings around the raised center; orange and red discoloration patterns on fruits; tip necrosis; and malformations [36,37].

Humans and animals are highly exposed to phytoviruses. In fact, plant viruses have been detected in humans, animals and even in environmental samples, including soil, water, cloud and fog [38,39]. The general belief is that phytoviruses infect only plants, and thus they do not represent any potential pathogenic threats to humans. However, this was recently reconsidered because a growing body of evidence is accumulating on cases when plant viruses were found to be able to replicate in animal cells too [40]. Moreover, the high similarity in genome organization between plant and animal viruses raises the question if some plant viruses could cross the kingdom border to cause diseases in humans or animals. For example, TSWV was shown to alter the male thrip feeding behavior, suggesting that it could be pathogenic also to the insect [8]. The potential pathogenicity of plant viruses in humans or other mammals remains a goal of further investigations.

Extracellular vesicles (EVs) and virus particles share several physical properties and mechanisms for biogenesis/viral exit and cellular uptake. Viruses subvert many host cell processes for their own replication, including the generation of EVs that carry fragments of viral genomes and viral proteins [41,42,43]. In addition, a majority of the techniques and methods for the isolation and characterization of EVs and plant NVs are the same as the ones used in virology research [44]. Another interesting point is that both modified plant viral nanoparticles and EVs are under development for targeted drug delivery and the development of vaccines [23,45,46].

**Figure 1:**
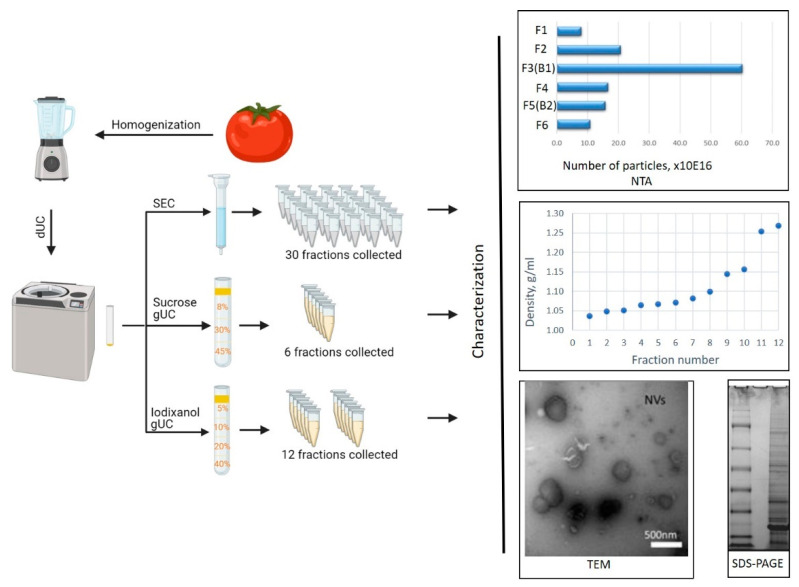
Isolation, physical and molecular characterization of tomato nanovesicles (NVs). Briefly, tomatoes were homogenized in a homogenization buffer, and differential centrifugation (dUC) was applied to isolate the bulk NVs. NVs were purified by size-exclusion chromatography (SEC) or separated into different fractions, using gradient-density ultracentrifugation (gUC). Particle concentration, size distribution, morphology, density and molecular features were analyzed to confirm the membrane vesicle character of the samples [14]. Inserts from top to the bottom show the particle number distribution in the sucrose gUC fractions performed by nanoparticle tracking analysis (NTA), density curve of the iodixanol gUC fractions, electron microscopy images and SDS–PAGE profile of crude tomato NVs. (Figure was created by using Biorender [47].)

In this work, we show that viral particles present in tomatoes homogenate of infected plant co-purify with NVs in the widely employed dUC or SEC isolation methods. To overcome this problem and to separate the viruses from the vesicles, we used gradient density ultracentrifugation (gUC). A combined characterization method relying on SDS–PAGE analysis, cryo-TEM and SEM morphological assessment and proteomics characterization was set up to detect and identify viruses (Figure 1).

## 2. Materials and Methods

### 2.1. Materials

Tomato fruits (Piccadilly variant) were purchased in the local market (G.M Fruit, Sicily, Italy). Sodium hydrogen phosphate dihydrate (Na_2_HPO_4_·2H_2_O), sodium dihydrogen phosphate (NaH_2_PO_4_·H_2_O) and sodium hydroxide (NaOH) were from J.T. Baker (Deventer, The Netherlands). Leupeptine, phenylmethylsulfonyl fluoride (PMSF), sodium azide, sucrose and colloidal Coomassie Brilliant blue G-250 were obtained from AppliChem (Darmstadt, Germany). OptiPrep™ (60% (*w*/*v*)) was from Serumwork (Bernburg AG, Germany). Sepharose Cl-2B, sucrose and Triton^®^ X-100 were obtained from Sigma-Aldrich, Inc. (St. Louis, MO, USA). RapiGest detergent was obtained from Waters Corporation (Milford, MA, USA). Trypsin (Mass Spec grade) was from Promega Corporation (Madison, WI, USA). Qubit Protein Assay Kit was from Thermo Fisher Scientific (Rockford, IL USA). Sodium dodecyl sulfate–polyacrylamide gel electrophoresis (SDS–PAGE) buffers, reagents and materials: Novex Bolt 4–12% Bis-Tris Plus gel and Bolt MOPS SDS running buffer were from Invitrogen (Carlsbad, CA, USA). Water (18.2 MΩ.cm (25 °C), 0.22 µm filtered) generated by a MilliQ system (Merck Millipore) was used. Other solvents used for proteomics were LC–MS grade (VWR International, Debrecen, Hungary). Leucine enkephalin peptide (amino acid sequence is YGGFL) was purchased from Waters Corp. (Wilmslow, UK).

### 2.2. Isolation of Tomato Nanovesicles by Differential Centrifugation

NVs were isolated from tomato fruits (250–300 g) by ultracentrifugation, as previously described [14]. Briefly, tomatoes were washed with MilliQ water, and exocarp was removed by placing the tomatoes into hot boiling water for a couple of seconds. Extraction buffer composed of 100 mM phosphate and 10 mM EDTA, pH 8 was added at 1:1 (*w*/*v*). Protease inhibitor cocktail containing 0.05 mL 1 mg/mL leupeptine, 0.25 mL 100 mM phenylmethylsulfonyl fluoride (PMSF) and 0.16 mL 1M sodium azide was added per every 100 g of tomatoes. The sample was homogenized by using the mixture grinder at maximum velocity for 10 s three times. Homogenized sample was centrifuged sequentially at 400× *g*, 800× *g* and 2000× *g*, using a swinging-out bucket rotor in an Eppendorf centrifuge 5804 R for 30 min, at 22 °C. Supernatant after the 2000× *g* step was centrifuged at 15,000× *g* in a 50 mL conical Eppendorf tube for 30 min, at 22 °C, using a fixed-angle rotor in an Eppendorf centrifuge 5804 R. The supernatant was subjected to ultracentrifugation at 100,000× *g* for 2 h, at 4 °C, in a SW28Ti rotor in a Beckman Coulter Optima L-90K ultracentrifuge. The pellet was solubilized in a small volume of extraction buffer, and protein concentration was measured by using the Qubit Protein Assay Kit.

### 2.3. Fractionation of Tomato Nanovesicles by Density Gradient Ultracentrifugation and Density Determination

Crude tomato NVs sample isolated by the differential centrifugation procedure was fractionated by dUC, using sucrose or OptiPrep (iodixanol) gradient solutions. Sucrose gUC was performed according to the procedure described in Reference [14], by under layering the sample with 8, 30 and 45% (*w*/*v*) sucrose cushions in a 38.5 mL polypropylene tube and centrifuging at 100,000× *g* for 2 h, at 4 °C, and by using a SW28Ti rotor (Beckman Coulter, Brea, CA, USA) in a Beckman Coulter Optima L-90K ultracentrifuge. Six fractions were collected from top to bottom. Sucrose was removed from the fractions by performing a washing ultracentrifugation step in the extraction buffer at 100,000× *g* for 1 h, at 4 °C. The resulting pellets were solubilized in a small volume of buffer, and protein concentration was measured by Qubit assay.

Iodixanol density gUC was performed in a 12.5 mL polypropylene ultracentrifugation tube (Beckman Coulter, Brea, CA, USA) layered by 5, 10, 20 and 40% (*v*/*v*) iodixanol (OptiPrep™) solution. Crude NV containing sample obtained by dUC was layered on the top of the gradient, and the sample was centrifuged at 100,000× *g* for 18 h, at 4 °C, using a SW41Ti rotor (Beckman Coulter, Brea, CA, USA). Twelve 1 mL volume fractions were collected from top to bottom. The fractions were washed in the extraction buffer by ultracentrifugation at 100,000× *g* for 1 h, at 4 °C, to remove the iodixanol. After washing, the pellets were solubilized in a small volume of buffer, and protein concentration was measured by the Qubit assay.

The density of the fractions was determined based on their iodixanol concentration, using standard absorption curve measured by using 5 different concentrations of the OptiPrep solution in water between 5 and 40% (*v*/*v*). Iodixanol gUC fractions were diluted at a ratio 1:5000 in MilliQ water, and the absorbance was measured at 244 nm wavelength by Nanodrop 2000 spectrophotometer (Thermo Fisher Scientific Inc., Waltham, MA, USA), according to manufacturer’s instruction. Based on their absorbance, the iodixanol percentage and the density of the fractions were calculated.

Iodixanol- and sucrose-density gUC fractionations were performed several times, using different dUC NV isolates.

### 2.4. Size-Exclusion Chromatography of Tomato-Derived Nanovesicles

SEC purification of dUC isolated crude tomato NV samples was carried out as previously described [14]. A 5 mL bed volume gravity column packed with Sepharose Cl-2B was used. Sample (200 µg in 250 µL extraction buffer) was loaded on the top of the column. Extraction buffer diluted ten times was used as an elution buffer. Thirty fractions of 250 µL each were collected. Protein concentration was measured in each fraction by the Qubit assay. After chromatography, the column was cleaned by 10 volumes of elution buffer, followed by 1 volume 1% (*v*/*v*) Triton, 1 volume of 0.5 M NaOH and 10 volumes of elution buffer.

### 2.5. Determination of Physical and Molecular Characteristics of Tomato-Derived NVs

Particle size distribution and particle concentration were measured as described earlier [14]. SDS–PAGE was performed as described in Reference [14] to separate proteins and to analyze the protein profiles. Samples (10 µg measured as protein content by the Qubit assay) were loaded and separated on a Novex Bolt 4–12% Bis-Tris Plus gel, using Bolt MOPS SDS running buffer and applying 150 V for 45 min. Gel was stained with colloidal Coomassie Brilliant blue G-250 overnight and washed with MilliQ water until the background was clear to view.

### 2.6. Cryogenic Transmission Electron Microscopy (Cryo-TEM)

Samples for the cryo-TEM were prepared by using the Vitrobot Mark IV (Thermo Fisher Scientific, Waltham, MA, USA). Quantifoil^®^ R 2/2, 200 (Quantifoil Micro Tools GmbH, Großlöbichau, Germany) or C-Flat 2/2, 200 mesh (Protochips, Morrisville, NC, USA) holey carbon grids were glow discharged for 60 s at 20 mA and positive polarity in air atmosphere (GloQube^®^ Plus, Quorum, Laughton, UK). Vitrobot conditions were set to 4 °C, 95% relative humidity, Blot time 3 s and Blot force 1. Then, 2 µL of the suspension was applied to the grid, blotted and vitrified in liquid ethane. Excess liquid was removed by filter paper. Samples were visualized with a 200 kV microscope Glacios with a Falcon 3EC detector (Thermo Fisher Scientific, Waltham, MA, USA).

### 2.7. Scanning Electron Microscopy (SEM)

Samples were prepared for SEM by a protocol adopted from Reference [48]. Samples were incubated for two hours in 2% OsO_4_ and dehydrated in a graded series of ethanol (30–100%), followed by a graded series of hexamethyldisilazane (mixed with absolute ethanol; 30%, 50% and 100%), and finally air dried. The dehydrated samples were coated with gold and palladium and examined by JSM-6500F Field Emission Scanning Electron Microscope (JEOL Ltd., Tokyo, Japan).

### 2.8. LC–ESI–MS/MS Analysis

In-gel and in-solution digestion proteomics were performed to identify proteins. For the in-solution digestion, samples were lysed in 0.2% RapiGest detergent and using 5 freeze–thaw cycles in liquid nitrogen and under sonication. After lyses, vesicles were digested, using trypsin: total proteins 1:50 ratio. For the in-gel digestion, selected protein bands were excised from the polyacrylamide gel, reduced and alkylated and digested with trypsin, according to Reference [49]. Prior to mass spectrometry (MS) analysis, samples were dissolved in 2% acetonitrile containing 0.1% (*v*/*v*) formic acid. MS experiments were performed on a high-resolution hybrid quadrupole-time-of-flight mass spectrometer (Waters Select Series Cyclic IMS, Waters Corp., Wilmslow, UK) equipped with a low flow electrospray ionization source. Chromatographic separation was carried out by using a Waters Acquity I-Class UPLC system on-line coupled to the mass spectrometer. A Waters Acquity CSH Peptide C18 UPLC column (1.7 µm, 1 mm × 150 mm) was used for chromatographic separation of the peptides. Gradient elution was performed with the following parameters: eluent A, 0.1% formic acid; eluent B, 0.1% formic acid in acetonitrile; flow rate, 20 µL/min; column temperature, 45 °C; gradient 5% B, 0–1 min, 5–35% B, 1–45 min, and 85% B, 45–46 min. MS data acquisition was performed with the following parameters: *m*/*z* 50–2000, V-mode, scan time of 0.5 s and single Lock Mass = leucine enkephalin. Fragmentation was performed in the trap: low energy: 6V, high energy: ramping 19–45 V. ProteinLynx Global Server 3.0.3 (Waters Corp., Wilmslow, UK) was used for data analysis. Background noise was filtered by using the Compression Tool 1.10 (Waters Corp., Wilmslow, UK); the threshold was set to 10 ion counts. Processing parameters were the following: low energy threshold, 200 counts; elevated energy threshold, 20 counts; minimum fragment ion matches per peptide, 3; minimum fragment ion matches per protein, 7; minimum peptide matches per protein, 2. The UniProt database was used with *Solanum lycopersicum* taxonomy ID 4081 (SOLLC containing 39020 sequence entries and including proteins from common tomato viruses).

### 2.9. Bioinformatics

FASTA format of the identified proteins was built by the Retrieve/ID mapping tool of UniProt. Functional annotation was performed by using the OmicsBox 1.4.12 [50] software. The Blastp search algorithm was used via NCBI web service without taxonomy filter, number of Basic Local Alignment Search Tool (BLAST) hits 20 and expectation value 1.0 × 10^−3^. The InterPro domain searches were performed by using the same input FASTA file. The BLAST hits of each protein sequence were mapped with Gene Ontology (GO) terms deposited in the GO database. Orthology assignment and clusters for orthologous groups (COGs) annotation were performed by the built-in EggNOg Mapper, using all target orthologues.

## 3. Results

### 3.1. Isolation and Characterization of Tomato-Derived Nanovesicles

We isolated NVs from different batches of commercial tomatoes (Piccadilly variant), using our previously described [14] dUC protocol. Generally, a high yield of crude NVs was obtained both in terms of number of particles (3.8 × 10^16^ particles per kilogram of fruit) and amount of proteins (26 ± 11 mg of proteins per kilogram of fruit).

**Figure 2:**
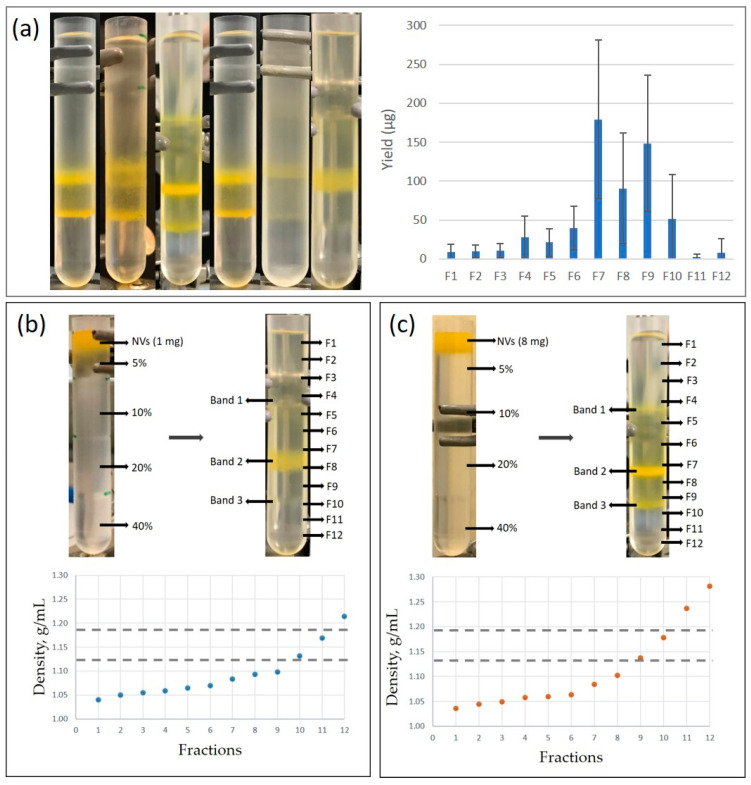
(**a**) Separation of nanovesicles (NVs) from different batches of tomatoes (Piccadilly cultivar) by iodixanol density gradient ultracentrifugation (gUC) (1 mg of NVs loaded, *n* = 6); graph on the right shows the average yields based on protein concentration in each fraction. (**b**) Iodixanol gUC of bulk tomato nanovesicles (NVs) without viruses, and (**c**) iodixanol gUC of bulk tomato NVs containing viral particles. Density curves show the densities calculated in each fraction. Horizontal dashed lines indicate the density range typical of mammalian extracellular vesicles [51].

**Figure 3:**
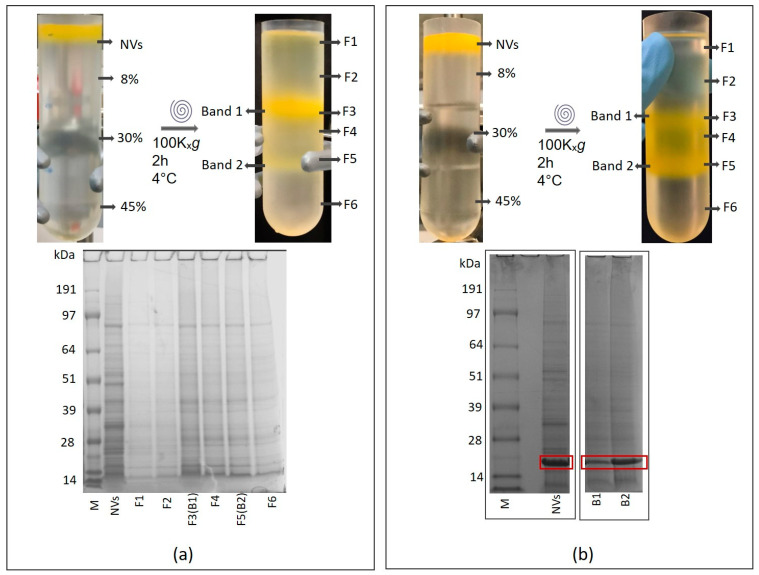
Gradient density ultracentrifugation using 45, 30 and 8% (*w*/*v*) of sucrose cushions and SDS–PAGE images of (**a**) bulk tomato nanovesicles (without viruses) and (**b**) bulk tomato nanovesicles containing viral particles.

Crude NVs were further purified by SEC or separated into different fractions, using gUC (Figure 1). GUC was performed by using iodixanol (Figure 2) or sucrose (Figure 3) as gradient solutions. Purified vesicles and fractions were analyzed by particle size distribution, morphology and SDS–PAGE protein profiles. Figure 2a shows the typical separation of bulk NVs into 12 fractions on iodixanol gradient performed on six different isolates. Generally, there were two—sometimes three—visible yellow colored bands that were collected in Fractions 4 (F4), 7 (F7), 8 (F8) and 9 (F9). The densities of the three visible bands from top to down were 1.070 ± 0.011 g/mL, 1.089 ± 0.007 g/mL and 1.12 ± 0.023 g/mL (Figure 2b). The densities of the two lower fractions were somewhat lower than the density range reported for mammalian EVs (1.13–1.19 g/mL) [51].

A typical result of the separation of NVs on a sucrose-cushion-density gradient is shown in Figure 3a. Sucrose density gradient material is widely used for the isolation of both EVs and viruses. Separation of tomato NVs on sucrose cushions, similarly to the iodixanol gradient, led to two and sometimes three visible bands in the low-density (B1) and high-density (B2) regions (Figure 3a).

**Figure 4:**
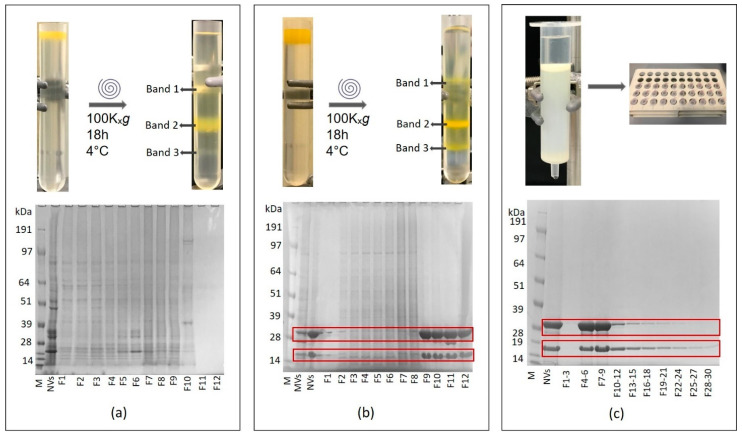
Sodium dodecyl sulfate–polyacrylamide gel electrophoresis protein profiles of (**a**) bulk tomato nanovesicles (NVs) without virus and the density-separated fractions F1–F12, (**b**) bulk tomato NVs containing viral particles and density separated fractions F1–F12 and (**c**) size-exclusion chromatography of virus containing NVs. NVs typically elute at SEC F4-6.

We used SDS–PAGE analysis to follow-up with the NV purification process. SDS–PAGE gel image of crude NVs isolate usually shows a complex but reproducible protein profile (Figure 3a and Figure 4a). Occasionally, we observed unusual SDS–PAGE profiles characterized by the presence of one or two intense Coomassie-stained protein bands in the low-molecular-mass region of the gel (Figure 3b and Figure 4b). These bands were not present in the SDS–PAGE gel of the majority of the batches. We used SEC as an attempt to remove the co-purifying protein “impurities” from the vesicle samples (Figure 4). After SEC, however, the intense low-molecular-mass bands were still present in the SDS–PAGE gel of SEC Fractions 5–9, where typically NVs elute (Figure 4c). As a second attempt, we applied the density-based gUC to remove the “impurity” from the samples. We analyzed gUC fractions by SDS–PAGE. Figure 4b shows that, based on the SDS–PAGE profile, gUC effectively separated the tomato NVs (in Fraction 4–7) from the low-molecular-mass “impurities” (in Fractions 9–12).

### 3.2. Cryo-TEM Analysis Shows Viral Contamination in some Tomato Nanovesicles Samples

**Figure 5:**
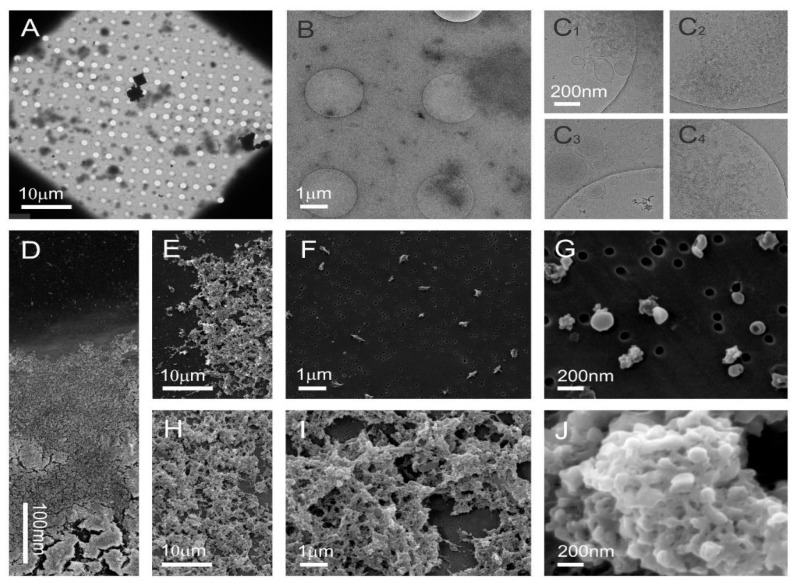
Cryo-TEM and SEM images of sucrose density separated nanovesicles in the low-density visible Fraction B1 isolated from tomatoes homogenate associated with an SDS–PAGE profile (Figure 3a) that did not show the presence of viral proteins. Cryo-TEM images (**A**–**C**) and SEM images (**D**–**J**) show the sample rich with sub-micron sized particles that are heterogeneous in size and shape, and numerous membrane-enclosed vesicles.

Cryo-TEM images of the sucrose density separated visible band B1 of tomato NVs (Figure 5A–C, Figure S1) reveal that the sample was rich in sub-micron sized particles of different sizes and shapes (Figure 5B,C, Figure S1). This sample showed an SDS–PAGE profile characteristic to NVs without the “contaminating” bands in the low-molecular region (Figure 3a). Some of these particles were delimited by the double-layered membrane and had smooth contours. Such shapes are characteristic for entities without internal structure that are enclosed by the membrane (Figure 5B,C). SEM images provide further evidence in agreement with this. Amorphous material observed in Figure 5(C_1_,C_2_,C_4_),D,E,H,I could correspond to cell fragments formed during processing of tomatoes and to already formed granular matter in the cells that is released into the exterior upon the rupture of the cell due to mechanical force. Alternatively, the amorphous material could be the vesicles which fused during the SEM sample preparation process (Figure 5J).

**Figure 6:**
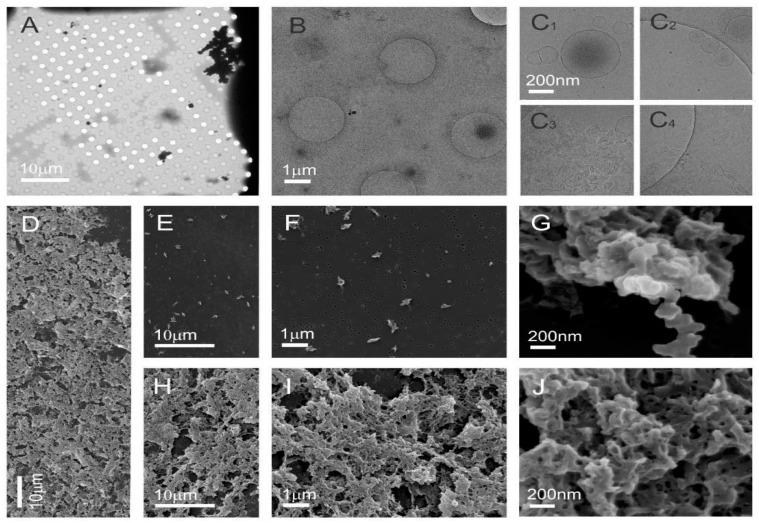
Cryo-TEM and SEM images of high-density B2 fraction of nanovesicles isolated from the tomato homogenate. Cryo-TEM images (**A**–**C**) and SEM images (**D**–**J**) show singular irregularly shaped particles and agglomerates.

Figure 6 and Figure S2 show cryo-TEM and SEM images of the high-density band B2 separated from NVs isolated from tomatoes homogenate (Figure 3a) and Figure S3 shows the SEM images of the SEC purified crude NVs These samples were also rich with sub-micron sized particles of different sizes and shapes. Cryo-TEM reveals the presence of vesicles; however, their proportion with respect to amorphous particles appeared lower. In SEM images, the presence of vesicles is indicated in agglomerates (Figure 6G, Figure S3).

**Figure 7:**
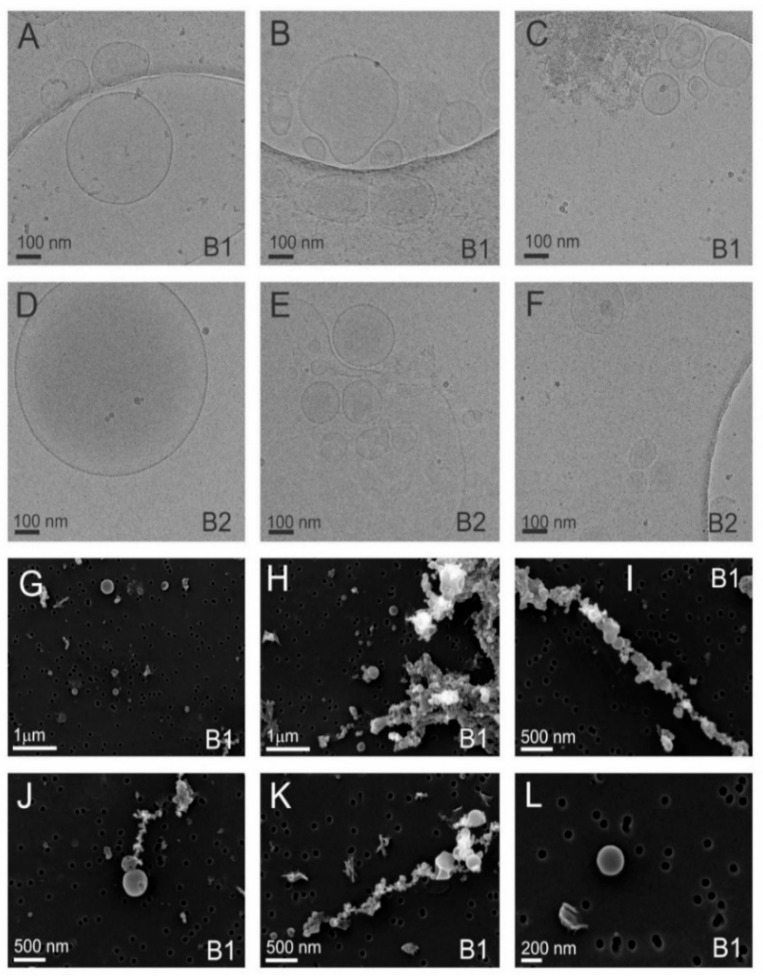
Cryo-TEM and SEM images of selected nanovesicles in the low (B1)—and high (B2)—density sucrose gradient-separated samples isolated from the tomatoes homogenate. Cryo-TEM images (**A**–**F**) show the presence of amorphous material as well as of vesicles enclosed by a bilayer membrane. SEM images (**G**–**L**) show the presence of particles with smooth shape that are characteristic for membrane-enclosed entities without internal structure—i.e., vesicles.

Selected details from the two main sucrose density separated Fractions B1 and B2 are shown in Figure 7. As ice in the cryo-TEM technique is about 100 nm thick, larger vesicles are squeezed into an oblate ellipsoid, which appears circular from the top (Figure 6(C_1_) and Figure 7A,D,E). The membrane bends in order to avoid contact with neighboring vesicles (Figure 5(C_3_) and Figure 7B). In some cases, the vesicles seem to adhere to one another and form a double bubble (Figure 6(C_1_) and Figure 7E). The inside of most vesicles is darker than the surrounding liquid indicating that the material is trapped inside the vesicles (Figure 5B,(C_1_), Figure 6B,(C_1_,C_2_) and Figure 7A–F). Some contours of vesicles contain contours of smaller vesicles indicating possibilities that the smaller vesicle is inside the larger one—or that the smaller vesicle is above or below the larger one when the freezing started and was dragged into the central part of the larger flaccid vesicle when that one was squeezed. With cryo-TEM we obtained crucial information on the morphology and morphology-based identification of particles in the samples and in particular on the proportions of different types of particles. Also we observed electron-dense material inside some vesicles. With SEM we observed three-dimensional shapes of vesicles, which were spherical, stomatocytic (Figure 5G, Figure 6G and Figure 7G–L) and globular with multiple invaginations (Figure 7J). These shapes correspond to the minimal free energy of the membrane and characterize entities in which the membrane encloses fluid interior [52]. Visualizing such shapes shows that the particles in question are indeed the membrane—enclosed vesicles. However, in the images of the UC isolate the majority of structures seem to derive from mechanically driven fragmentation of cells. The double layer around these particles is not observed and the shapes should be determined by the internal structure of the particles. The particles that do not have smooth contour (Figure 5(C_2_,C_4_), Figure 6(C_3_,C_4_) and Figure 7B,C) are expected to be protein and lipoprotein aggregates. Vesicles and these particles readily interact between each other thereby forming a line-up (Figure 7H–K) or larger aggregates (Figure 5J and Figure 6G).

As we did not observe many vesicles in B2 in the SEM images, there is a possibility that they were destroyed during the SEM preparation, which is more aggressive than preparation for cryo-TEM. Combination of cryo-TEM and SEM of the same sample therefore provides complementary data, which help in building an interpretation of the content of the sample.

**Figure 8:**
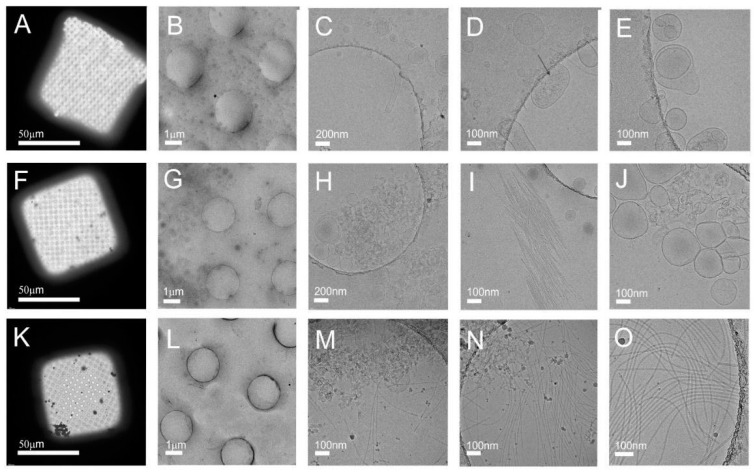
Cryo-TEM images of three fractions of isolate from the homogenate of tomato infected by the virus: Fraction 4 (**A**–**E**), Fraction 7 (**F**–**J**) and Fraction 9 (**K**–**O**). Fraction 4 exhibits mostly EVs; Fraction 7 exhibits sub-micron-sized particles, vesicles and virions; and Fraction 9 exhibits mostly virions.

Figure 8 shows cryo-TEM images of three fractions of tomato NVs separated by iodixanol gradient ultracentrifugation (Figure 4b and Figure S4): Fraction 4 (Figure 8A–E), Fraction 7 (Figure 8F–J) and Fraction 9 (Figure 8K–O). A high number of vesicles can be seen in Fraction 4 (Figure 8C–E). A rod-like structure is enclosed within the vesicle contour in Figure 8D (black arrow), indicating a possibility that the virions or their fragments are contained in the vesicle. However, in the same picture, there is a filamentous structure outside the vesicle contour too. Fraction 7 exhibits nanovesicles (Figure 8G–J), cellular fragments (Figure 8H,J) and also viral particles (Figure 8I,J). Although all three types of particles can be found also in Fraction 9, virions largely prevail in Fraction 9. This can be seen by comparing enlarged Figure 8B,G,L. The results show that Fraction 9 is very rich in viral particles.

### 3.3. Proteomics Reveals the Identity of Viral Particles-Related Proteins in Tomato-Derived Nanovesicles

LC–ESI–MS/MS-based shotgun proteomic analyses were performed on the gUC fractions of two NVs isolates that showed unusual SDS–PAGE profiles. In the first iodixanol gradient-separated sample (Figure 4b), three distinct visible gUC bands (Fraction 4, Fraction 7 and Fraction 9) and three abundant SDS–PAGE bands of Fraction 9 (Figure 2c, F9_band1, F9_band2 and F9_band3) were analyzed. In the second sample, separated on sucrose gradient, two visible bands (Band 1 and Band 2, not shown) were analyzed.

#### 3.3.1. Sample 1 Contains Tomato Vesicles and Three Different Viral Particles

177 proteins were identified in low-density Fraction 4 of sample 1 (Supplementary Materials Table S1). The twenty top-ranking proteins are reported in Table 1. Since several of the identified proteins have not yet been characterized, functional annotation was performed. The first protein in Table 1 is an uncharacterized protein (A0A3Q7IXE6_SOLLC) that has a lipid binding domain. It shows high structural similarities to Patellin-3 (PTL3) protein. In *Arabidopsis*, PTL3 is involved in membrane-trafficking events associated with cell-plate formation during the reproductive and vegetative development process. Interestingly, PTL3-like protein was also found to be highly expressed in citrus-fruit-juice-derived micro and nanovesicles [4]. Other highly expressed proteins in this fraction (Table 1) were the subunits of different V-ATPases (Q84XW6_SOLLC, Q84XV7_SOLLC, A0A3Q7FE06_SOLLC and A0A3Q7IIS5) and plasma membrane ATPases (Q9SPD5_SOLLC, PMA1_SOLLC and K4DFV3_SOLLC), heat-shock proteins (A0A3Q7IZ03, A0A3Q7FX57 and A0A3Q7IYI9) and 14-3-3 protein (A0A3Q7EZ16), all typically expressed in edible plant-derived vesicles. Lipoxygenases (LOXs; P38416 and A0A3Q7ENA3) were also highly expressed in this fraction. LOX is a ubiquitous enzyme in the animal and plant kingdoms, and it was found to be abundant in previous tomato NV preparation [14]. LOXs act on natural polyunsaturated fatty acids, such as linoleic acid and arachidonic acid, and catalyze the formation of corresponding hydroperoxides. The role of LOXs in growth, development and response to stress to pathogen infection and wound is emerging, although not yet completely clarified. Besides LOXs, we have identified another four proteins involved in the defense response to other organisms (GO: 0098542), using a bioinformatics approach based on GO-term analysis. These are knot 1 domain containing proteins (A0A3Q7H3Y0), LEA-2 domain containing protein (A0A3Q7ILU4) and two Pto-interacting proteins (Q41328 and A0A3Q7GN48) (Supplementary Materials Table S1). GO-term analysis of the proteins identified in Fraction 4 shows that the transmembrane transport cellular process sub-GO-term (GO: 0055859 of cellular processes was enriched, which suggests an enrichment in transport function related to vesicles (not shown).

Interestingly, besides the proteins of *S. lycopersicum*, two viral proteins could also be identified in Fraction 4. These were the capsid proteins from brown rugose fruit virus, isolate ToBRFV/Tomato/Jordan/Tom1-Jo/2015) (A0A0S2SZX3) and the capsid protein from the ToMV, Korean strain (Q83482) (Supplementary Materials Table S1). The identification of viral proteins in the vesicle fractions were concordant with the results obtained by cryo-TEM and confirms that, even though Fraction 4 is highly enriched in vesicles, it is not completely free of viruses (Figure 8D).

The capsid protein of ToBRFV is the first ranking protein in Fraction 7 of sample 1, suggesting that ToBRFV virus is rich in this fraction. Several *S lycopersicum* proteins (36 proteins) were also identified, and they show an overlap to proteins identified in Fraction 4. However, the fact that fewer proteins were identified indicates that fewer vesicles are present in this fraction than in Fraction 4. Moreover, the capsid protein of ToMV identifies the presence of a second virus in this fraction. Since ToMV is a rod-shaped virus with similar dimensions as ToBRFV, their co-isolation in the same fraction is reasonable.

The capsid protein of the ToBRFV virus was also expressed in Fraction 9 of sample 1. This fraction contains mainly this virus, and it was further confirmed by in-gel digestion proteomics of the three bands excised from SDS–PAGE (F9_band1, Fr9_band2 and Fr9_band3). In band 3, besides ToBRFV, another virus, ToMMV, was also identified by its capsid protein.

#### 3.3.2. Sample 2 Contains Tomato Vesicles in the Low-Density and Viral Particles in the High-Density Sucrose Fractions

The low-density sucrose fraction of sample 2 (B1) was characterized by a high number of *S. lycopersicum* proteins (994 proteins; Supplementary Materials Table S1), indicating that tomato-derived NVs prevail in this fraction. However, this fraction was not completely free of viral proteins, since proteins of the potato virus Y (PVY), southern tomato virus (STV) and TSWV could be detected (Supplementary Materials Table S1) too.

The high-density sucrose fraction of sample 2 (B2) was abundant in proteins of TSWV but contained also vesicle-related proteins, confirming an incomplete separation between viral particles and NVs in the sucrose gradient separation. Almost the whole proteome, i.e., four out of five TSWV proteins, could be identified in this fraction: capsid protein (glycoprotein), putative movement protein, nucleoprotein and the NSs non-structural protein. The presence of many viral proteins in B2 suggests that this fraction could be enriched in intact TSWV particles. It is of note that TSWV is very similar in size and morphology to vesicles but has a higher density (1.207 g/mL). Interestingly, one protein, the genome polyprotein of PVY, was also identified in B2; however, the presence of this virus in this fraction appeared to be less prominent than in B1.

Table 2 summarizes different viruses and their proteins identified in the gUC fractions of two tomato NV samples by proteomics.

## 4. Discussion

Several recent studies focus on the isolation and characterization of nanometer-sized membrane-enclosed vesicles from whole plant [1,9] or different organs, such as the root [56], seed [57], fruits [1,2,3,5,14,20,23,57], stems or leaves [22]. These biomembrane-enclosed structures were reported to be similar to mammalian EVs in their morphological and physical characteristics [14,57,58,59]. Isolation of NVs typically starts with the homogenization of the plant material which is composed of different tissues and cell types. Homogenization leads to the rupture of the cell membrane resulting in the release of the cell content. Consequently, an NV sample isolated from plant material typically contains a very heterogeneous and dynamic mixture of intra and extracellular vesicles, as well as vesicles that formed in the isolation process. NVs isolated from plants were shown to be efficiently uptaken by recipient cells, and they were associated with anticancer, anti-inflammatory and antioxidative effects. Moreover, recent research focuses on their exploitation in molecular delivery and tissue regeneration [3,7,11,15,16,17,18,19,20,21,22,23].

Some of the crude tomato NV samples (dUC isolates) showed an unusual SDS–PAGE profile with prominent bands at the low molecular region (Figure 4b). The combination of SDS–PAGE profiling with cryo-TEM and SEM-based morphological study and proteomics analysis revealed that these samples contained tomato viruses that co-purified with the nanovesicles in dUC. Tomato-derived NVs are spherical structures, have a 110 ± 10 nm size distribution measured at maximum by dynamic light scattering [14] and are characterized by a low buoyant density of 1.070–1.120 g/mL. On the other hand, most of the known plant viruses are filamentous with coat proteins forming a tube surrounding the viral genome and have a density in the range of 1.35–1.4 g/mL [60]. Due to these differences in densities, we showed that the buoyant density-based ultracentrifugation is able to separate plant NVs from viruses. We found that both sucrose and iodixanol gUC were indeed effective to enrich NVs in the lower-density fractions and viral particles in the higher-density fractions. In our hands, iodixanol gUC provided a better separation. However, it should be noted that we could not observe a complete separation and the low-density gUC fractions still contained some viral particles. The combined method used in this work turned out to be straightforward in the detection and identification of plant viruses whose genome sequences were available. In particular, electron-microscopic imaging could distinguish between rod-shaped virions from the round-shaped membrane-enclosed NVs. However, further studies are needed to ascertain if the method is capable of distinguishing spherical virions from the membrane vesicles.

Viral particles were identified based on their coat proteins or other viral proteome components by MS-based proteomics. In the two isolates studied here, we identified both commonly occurring viruses, such as ToMV, TSWV, PVY and STV, and emerging ones, such as ToBRFV and ToMMV (Table 2). Most of them were rod-shaped, with the exception of TSWV, which is roughly spherical shape. We cannot exclude the presence of other viruses whose genome information is unavailable.

EVs have recently emerged as a novel way of viral propagation exploited by both enveloped and non-enveloped viruses in mammals [43]. However, there is little known about vesicle-mediated viral exit and infection in plants [61]. Interestingly, we have identified viral proteins not only in the high but also in the low-density fractions where usually NVs are enriched. While this could be a consequence of their partial separation, at this point, we cannot exclude the hypothesis that the identified viral proteins or the intact virions could be secreted within the vesicles.

## 5. Conclusions

Here, we have shown that viruses in the fruit of tomatoes can co-purify with bulk membrane-bound nanovesicles in the dUC or SEC-based isolation protocols. SDS–PAGE was found to be useful to indicate the presence of viral contamination based on the unusual pattern due to the occurrence of abundant viral proteins at the low molecular mass. The combination of electron microscopy with mass spectrometry–based shotgun proteomics enabled us to distinguish viral particles by morphology and identify the virus by its proteins. Since dietary vegetables and fruits are foreseen to be increasingly applied in future medical, pharmaceutical and cosmeceutical applications as both active ingredient and nanosized delivery vector, it is important to develop methods that are able to remove virus particles from the vesicle isolates or to demonstrate that NV isolates are free of virus contamination. In this work, we demonstrated that gradient ultracentrifugation is a valuable method to separate the viral particles from the viruses when there is a difference in buoyant densities.

## Figures and Tables

**Table 1:** Top-ranking proteins’ functional annotations of tomato nanovesicles (NVs) isolated by iodixanol gradient density ultracentrifugation as low-density band (Fraction 4).

No.	Accession	Description UniProt	PLGS Score	Peptides	Coverage (%)	Description OmicsBOX (Protein Blast)	Sim Mean
1	A0A3Q7IXE6	Uncharacterized protein	14,872	47	79	patellin-3-like	94.26
2	Q84XW6	V-ATPase 69 kDa subunit	29,199	39	76	V-type proton ATPase catalytic subunit A	98.78
3	P38416	Linoleate 9S-lipoxygenase B	9129	32	44	putative linoleate 9S-lipoxygenase 5	90.26
4	A0A3Q7ENA3	Lipoxygenase	9055	31	43	putative linoleate 9S-lipoxygenase 5	90.1
5	Q84XV7	V-ATPase 69 kDa subunit	19,723	29	51	V-type proton ATPase catalytic subunit A	98.78
6	Q9XEX8	Remorin 1	10,015	22	51	remorin	86.7
7	A0A3Q7FE06	V-type proton ATPase subunit a	72,645	22	35	V-type proton ATPase subunit a3	95.26
8	Q9SPD5	Plasma membrane ATPase	6306	22	28	plasma membrane atpase 1	99.29
9	A0A3Q7IIS5	Vacuolar proton pump subunit B	24,212	21	62	V-type proton ATPase subunit B2	98.52
10	A0A3Q7IZ03	Uncharacterized protein	15,503	21	45	heat shock cognate 70 kDa protein 2-like	98.17
11	A0A3Q7FX57	Uncharacterized protein	15,788	20	42	heat shock cognate 70 kDa protein 2-like	99.12
12	A0A3Q7INZ6	Uncharacterized protein	44,097	18	48	actin-7	99.19
13	A0A3Q7GJM0	Phosphoinositide phospholipase	5957	18	38	phosphoinositide phospholipase C 2-like	95.35
14	A0A3Q7FJJ3	Uncharacterized protein	43,162	17	51	actin-7	99.87
15	A0A3Q7FRW6	PHB domain-containing protein	12,933	17	50	hypersensitive-induced reaction 1 protein	99.01
16	A0A3Q7EZ16	14_3_3 domain-containing protein	8764	17	58	14-3-3 protein 4	97.82
17	A0A3Q7IYI9	Uncharacterized protein	14,977	16	35	heat shock cognate 70 kDa protein	98.88
18	A0A3Q7FV11	H(+)-exporting diphosphatase	7652.922	16	13	pyrophosphate-energized vacuolar membrane proton pump-like	98.42
19	A0A3Q7I767	Fe2OG dioxygenase domain-containing protein	7169.406	16	37	1-aminocyclopropane-1-carboxylate oxidase homolog	83.31
20	A0A3Q7HFP1	Glycerophosphodiester phosphodiesterase	6081.231	16	22	glycerophosphodiester phosphodiesterase GDPDL4	88.91

**Table 2:** Viral proteins identified by proteomics in two tomato-derived nanovesicles samples (S1 and S2) and separated by gradient ultracentrifugation into fractions. * gUC fraction(s) where the viral protein(s) was (were) highly expressed based on protein ranking reported in Supplementary Materials Table S1.

Name of the Virus	Genus of the Virus	Viral Characteristics	Sample	gUC Fraction(s) *	Name of Viral Protein(s) Identified	UniProt Accession No.	Coverage % of Viral Protein(s) Identified
Tomato brown rugose fruit virus (ToBRFV)	*Tobamovirus*	Single-stranded RNA rod-shaped particles of 300 nm in length and 17 nm in diameter [31]	S1	4 7 9	Capsid protein	A0A0S2SZX3	55.3 54.7 55.4
Tomato mosaic virus (ToMV)	*Tobamovirus*	Single-stranded RNA rod shaped structure, about 300 nm length and 18 nm radius [27]	S1	4	Capsid protein	Q83482	6.4
Tomato mottle mosaic virus (ToMMV)	*Tobamovirus*	Single-stranded RNA rod-shaped virus particles 300 nm in length [28,29]	S1	7	Capsid protein	T1WEZ3	5.7
Tomato spotted wilt virus (TSWV)	*Orthotospovirus*	Single-stranded RNA roughly spherical shaped with a diameter 80–120 nm and density of 1.207 g/mL [34]	S2	B2	Nucleoprotein	O55648	58.1
					NSs non-structural protein	E1Y5V2	19.9
					Nucleocapsid protein	A0A0N9H8W3	56.7
					Putative movement protein	A0A097PIF5	30.1
Potato virus Y (PVY)	*Potyvirus*	Single-stranded RNA, a filamentous, flexuous form, with a length of 730 nm and a diameter of 12 nm [53,54]	S2	B1	Putative coat protein	A0A0K2K0B0	18.3
				B2	Genome polyprotein	P18247	9.3
Southern tomato virus (STV)	*Amalgavirus*	Double-stranded RNA, shape and size NA [55]	S2	B2	Putative coat protein	A0A0K2K0B0	18.3

## Data Availability

The data presented in this study are available on request from the corresponding author.

## Supplementary Materials

The following are available online at https://www.mdpi.com/article/10.3390/nano11081922/s1. Figure S1. A) Cryo-TEM and B) SEM images of sucrose density separated nanovesicles in the low-density visible fraction B1 isolated from tomatoes homogenate. Figure S2. (A) Cryo-TEM and (B) SEM images of sucrose density separated nanovesicles in the high-density visible fraction B2 isolated from tomatoes homogenate. Figure S3. SEM images of the SEC fractions isolated from the tomato homogenate. Figure S4. Cryo-TEM images of iodixanol density separated nanovesicles in three visible bands (Fraction 4, Fraction 7 and Fraction 9) isolated from the homogenate of tomato infected by the virus. Table S1: Proteomics results. In-solution digestion shotgun proteomics results of (A) iodixanol gradient ultracentrifugation Fraction 4, (B) iodixanol gradient ultracentrifugation Fraction 7 and (C) iodixanol gradient ultracentrifugation Fraction 9; (D) in-gel proteomics results of band 1 from the SDS–PAGE gel of Fraction 9, (E) in-gel proteomics results of band 2 from the SDS–PAGE gel of Fraction 9 and (F) in-gel proteomics results of band 3 from the SDS–PAGE gel of Fraction 9; (G) in solution proteomics results of a second batch of tomato NVs separated on sucrose density gradient in the low-density visible band and (H) in solution proteomics results of a second batch of tomato NVs separated on sucrose density gradient in the high-density visible band.
