# Supplementary material for: Identification of Tomato Infecting Viruses That Co-Isolate with Nanovesicles Using a Combined Proteomics and Electron-Microscopic Approach
Source: Nanomaterials (Basel). 2021 Jul 26;11(8):1922. doi: 10.3390/nano11081922 (PMC8399691; doi:10.3390/nano11081922)
Supplement: Supplementary file 1 [file nanomaterials-11-01922-s001.zip › Supplementary Figures S1-S4.pdf]

Supplementary Materials

Identification of Tomato Infecting Viruses That Co-Isolate with Nanovesicles Using a Combined Proteomics and Electron-Microscopic Approach

Ramila Mammadova [1], Immacolata Fiume [1], Ramesh Bokka [1], Veronika Kralj-Iglič [2], Darja Božič [2], Matic Kisovec [3], Marjetka Podobnik [3], Apolonija Bedina Zavec [3], Matej Hočevar [4], Gabriella Gellén [5], Gitta Schlosser [5] and Gabriella Pocsfalvi [1,*]

[1] Extracellular Vesicles and Mass Spectrometry Laboratory, Institute of Biosciences and BioResources, National Research Council of Italy, 80131 Naples, Italy; ramila.mammadova797@gmail.com (R.M.); immacolata.fiume@ibbr.cnr.it (I.F.); ramesh.chem2008@gmail.com (R.B.)

[2] Laboratory of Clinical Biophysics, Faculty of Health Sciences, University of Ljubljana, SI-1000 Ljubljana, Slovenia; veronika.kralj-iglic@fe.uni-lj.si (V.K.-I.); darja.bozic@fe.uni-lj.si (D.B.)

[3] Department of Molecular Biology and Nanobiotechnology, National Institute of Chemistry, SI-1000 Ljubljana, Slovenia; matic.kisovec@ki.si (M.K.); marjetka.podobnik@ki.si (M.P.); polona.bedina@ki.si (A.B.Z.)

[4] Institute of Metals and Technology, SI-1000 Ljubljana, Slovenia; matej.hocevar@imt.si

[5] MTA-ELTE Lendület Ion Mobility Mass Spectrometry Research Group, ELTE Eötvös Loránd University, Institute of Chemistry, H-1117 Budapest, Hungary; gabgellen@staff.elte.hu (G.G.); gitta.schlosser@ttk.elte.hu (G.S.)

* Correspondence: gabriella.pocsfalvi@ibbr.cnr.it

A)

B)

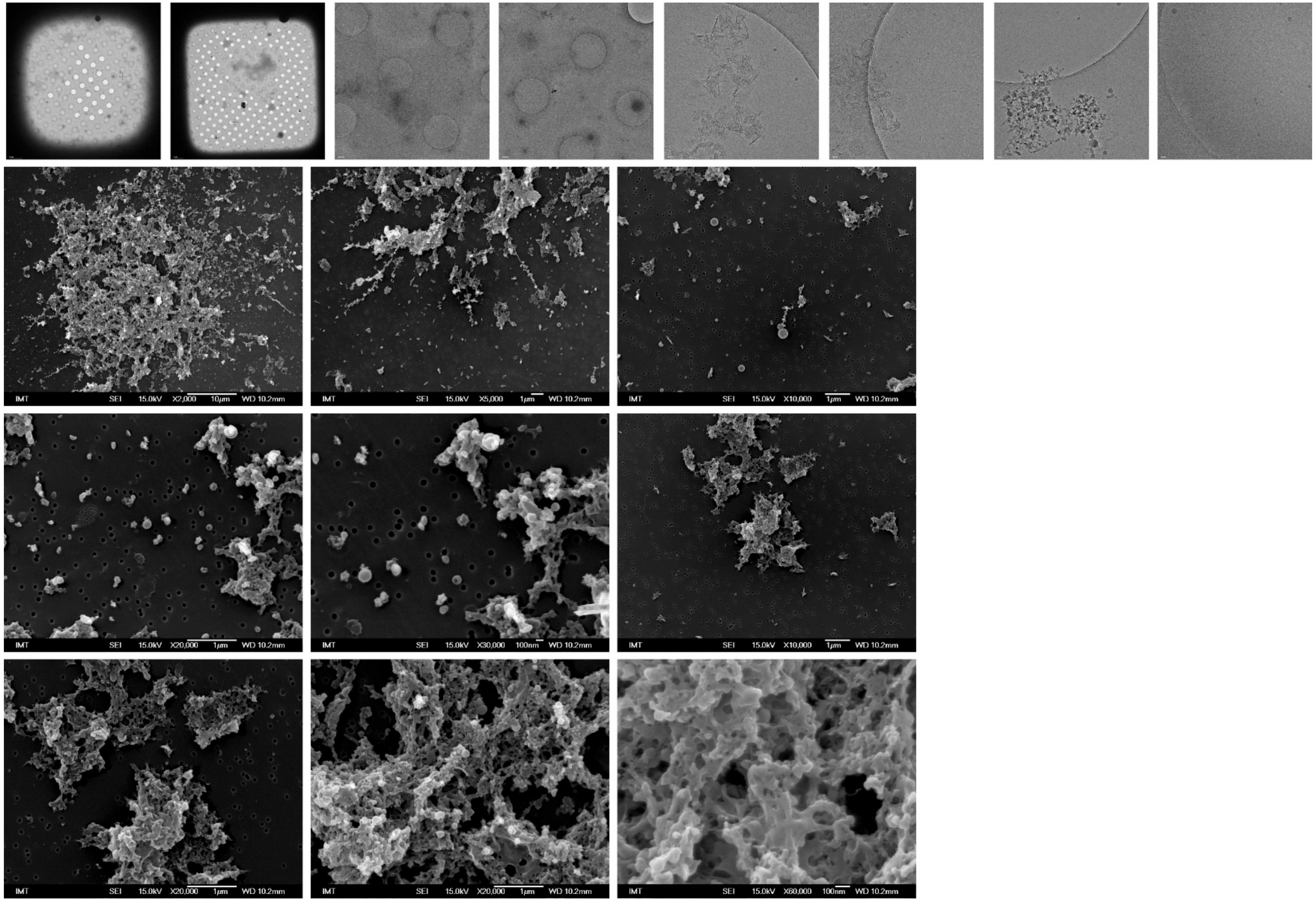

Figure S1. A) Cryo-TEM and B) SEM images of sucrose density separated nanovesicles in the low density visible fraction B1 isolated from tomatoes homogenate.

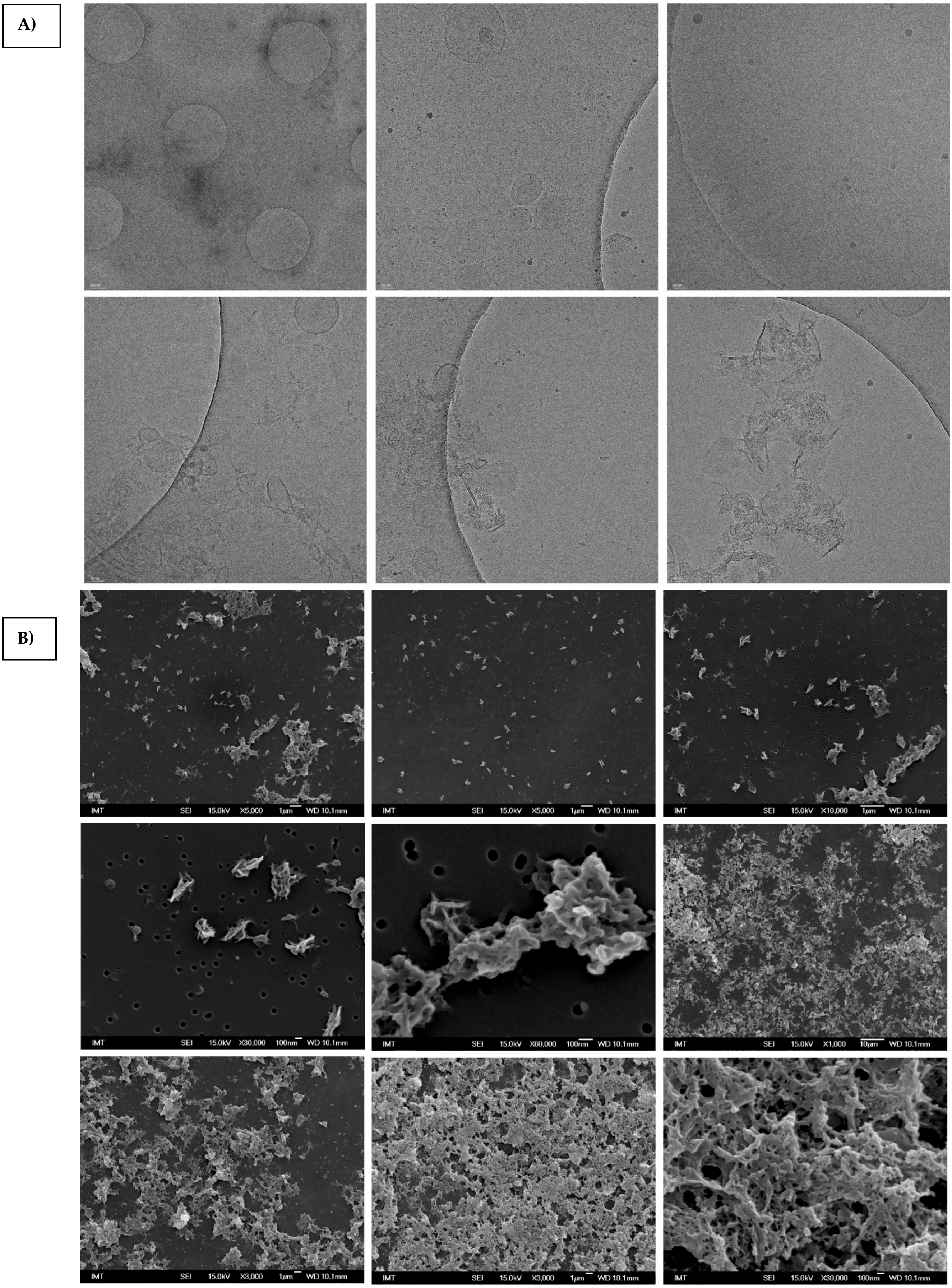

Figure S2. A) Cryo-TEM and B) SEM images of sucrose density separated nanovesicles in the high density visible fraction B2 isolated from tomatoes homogenate.

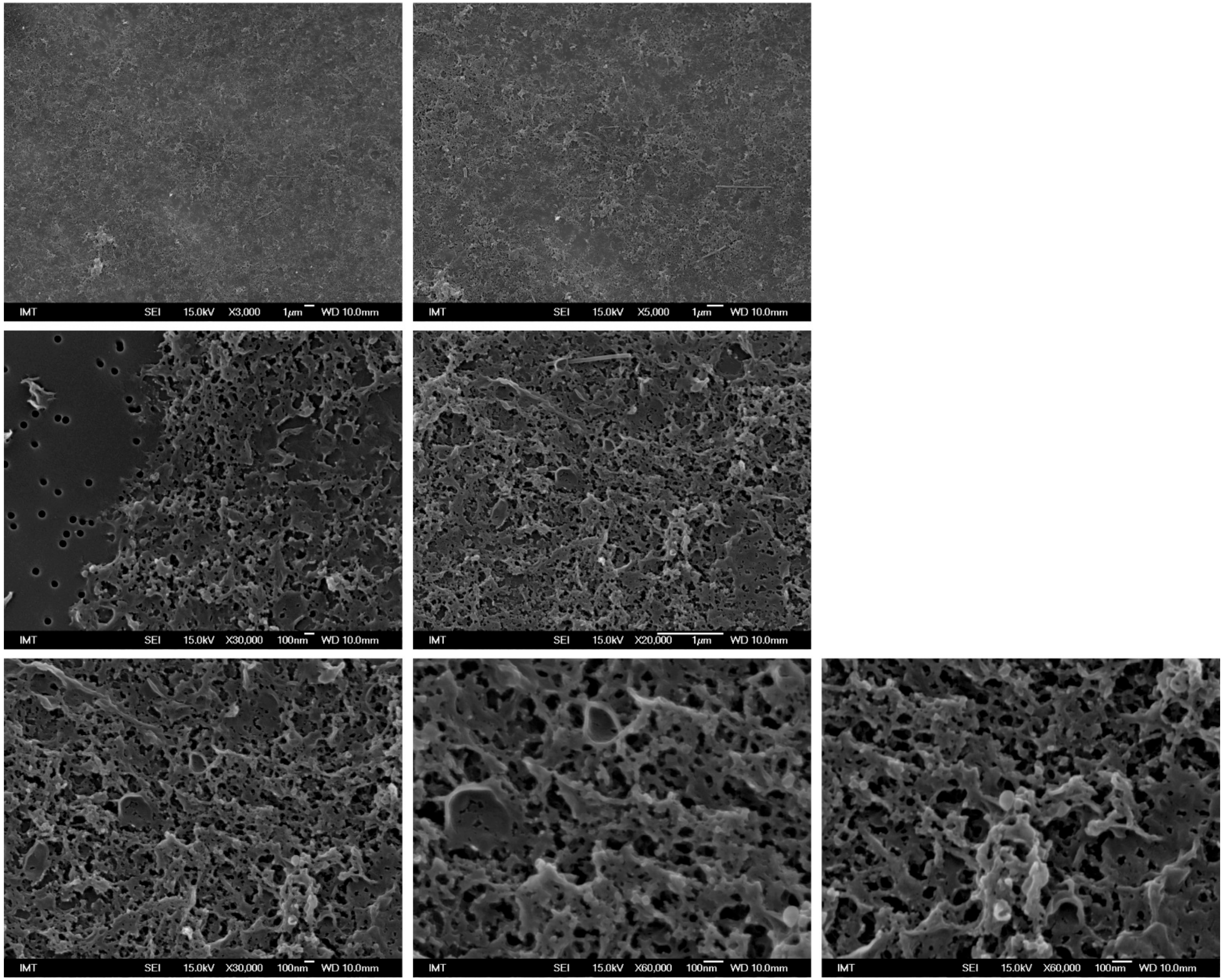

Figure S3. SEM images of the SEC fractions isolated from the tomato homogenate.

.

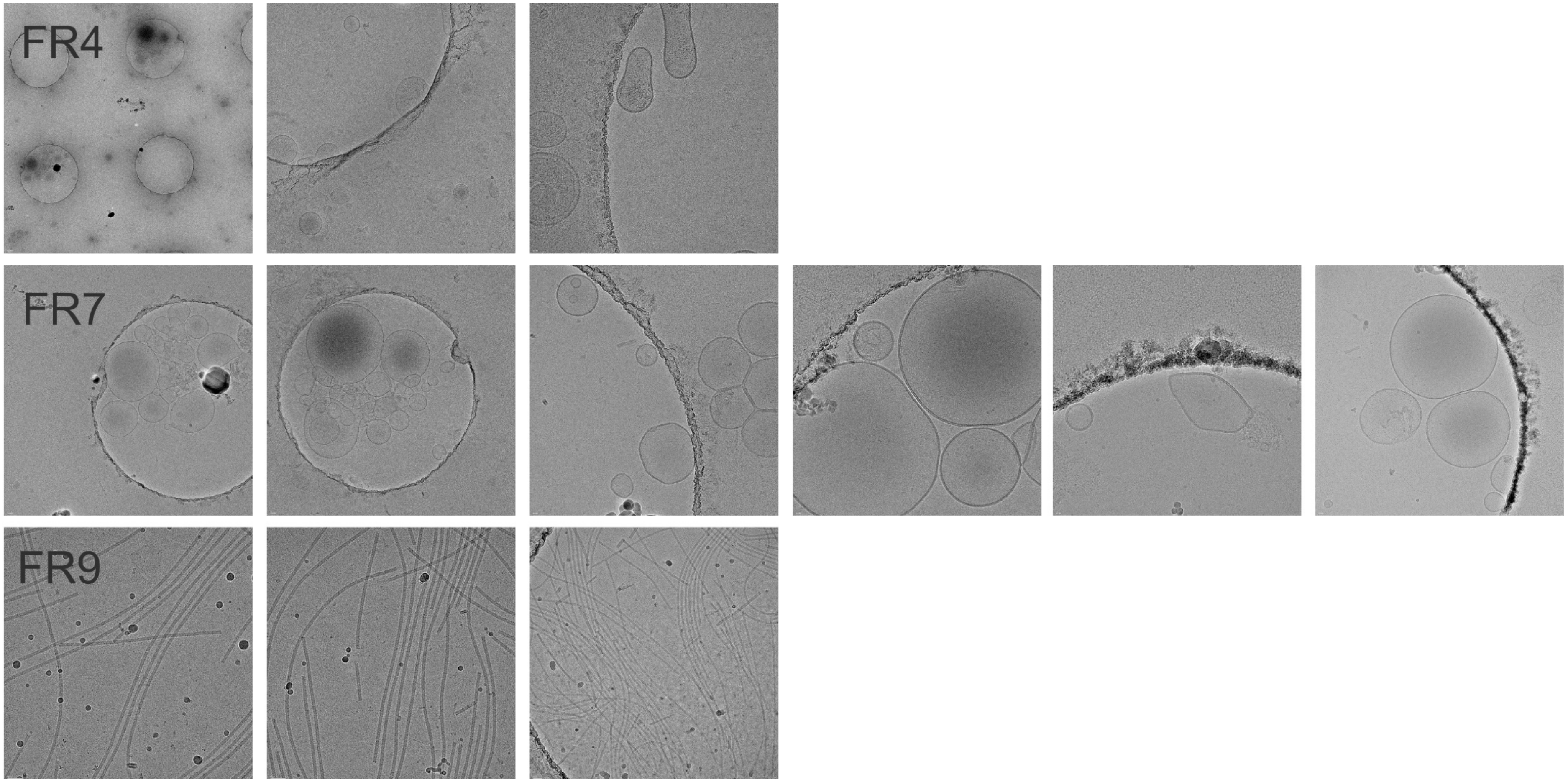

Figure S4. Cryo-TEM images of iodixanol density separated nanovesicles in three visible bands (Fraction 4, Fraction 7 and Fraction 9) isolated from the homogenate of tomato infected by the virus.
