# Supplementary material for: Identification of Tomato Infecting Viruses That Co-Isolate with Nanovesicles Using a Combined Proteomics and Electron-Microscopic Approach
Source: Nanomaterials (Basel). 2021 Jul 26;11(8):1922. doi: 10.3390/nano11081922 (PMC8399691; doi:10.3390/nano11081922)
Supplement: Supplementary file 1 [file nanomaterials-11-01922-s001.zip › Supplementary Table S1.pdf]

Row	Accession	Protein
1	P38416	Linoleate 9S-lipoxygenase B OS=Solanum lycopersicum GN=LOX1.2 PE=2 SV=1
2	O55648	Nucleoprotein OS=Tomato spotted wilt virus PE=2 SV=1
3	K4CWE4	Uncharacterized protein OS=Solanum lycopersicum PE=3 SV=1
4	V5M1X4	Nucleoprotein OS=Tomato spotted wilt virus GN=N PE=3 SV=1
5	V5M0P0	Nucleoprotein OS=Tomato spotted wilt virus GN=N PE=3 SV=1
6	Q65XZ3	Nucleoprotein OS=Tomato spotted wilt virus GN=N PE=3 SV=1
7	K4C227	Uncharacterized protein OS=Solanum lycopersicum PE=3 SV=1
8	K4D8S6	Uncharacterized protein OS=Solanum lycopersicum PE=4 SV=1
9	K4BVN6	Uncharacterized protein OS=Solanum lycopersicum PE=3 SV=1
10	K4BEL1	Uncharacterized protein OS=Solanum lycopersicum PE=3 SV=1
11	K4AV63	Uncharacterized protein OS=Solanum lycopersicum PE=3 SV=1
12	K4DAD5	Uncharacterized protein OS=Solanum lycopersicum PE=3 SV=1
13	K4C4Z6	Uncharacterized protein OS=Solanum lycopersicum PE=3 SV=1
14	A0A0N9H8W3	Nucleocapsid protein (Fragment) OS=Tomato spotted wilt virus PE=4 SV=1
15	Q0PTG1	Nucleoprotein OS=Tomato spotted wilt virus GN=N PE=3 SV=1
16	P14280	Pectinesterase 1 OS=Solanum lycopersicum GN=PME1.9 PE=1 SV=5
17	P28032	Alcohol dehydrogenase 2 OS=Solanum lycopersicum GN=ADH2 PE=2 SV=2
18	K4BL25	Uncharacterized protein OS=Solanum lycopersicum PE=3 SV=1
19	Q2VI27	Nucleocapsid protein (Fragment) OS=Tomato spotted wilt virus PE=4 SV=1
20	P18247	Genome polyprotein OS=Potato virus Y (strain N) PE=3 SV=2
21	Q53Z32	Molecular chaperone Hsp90-2 OS=Solanum lycopersicum PE=2 SV=1
22	Q6UJX4	Molecular chaperone Hsp90-1 OS=Solanum lycopersicum PE=2 SV=1
23	K4CEJ1	Uncharacterized protein OS=Solanum lycopersicum PE=4 SV=1
24	E3NYV4	Polyprotein OS=Potato virus Y (strain C) PE=3 SV=1
25	K4CSI2	Uncharacterized protein OS=Solanum lycopersicum PE=3 SV=1
26	A0A0C5CHE6	Ribulose bisphosphate carboxylase large chain OS=Solanum lycopersicum GN=rbcL PE
27	K4CQV5	Fructose-bisphosphate aldolase OS=Solanum lycopersicum PE=3 SV=1
28	Q8VXF8	Phosphoenolpyruvate carboxylase 2 OS=Solanum lycopersicum GN=ppc2 PE=3 SV=1
29	Q4LDR0	Heat shock protein OS=Solanum lycopersicum GN=Lehsp100/ClpB PE=2 SV=1
30	K4DFR1	Uncharacterized protein OS=Solanum lycopersicum PE=3 SV=1
31	K4BP30	Pyruvate kinase OS=Solanum lycopersicum PE=3 SV=1
32	K4BJW4	Glyceraldehyde-3-phosphate dehydrogenase OS=Solanum lycopersicum PE=3 SV=1
33	P38415	Linoleate 9S-lipoxygenase A OS=Solanum lycopersicum GN=LOX1.1 PE=2 SV=1
34	P10967	1-aminocyclopropane-1-carboxylate oxidase homolog OS=Solanum lycopersicum GN=
35	K4BYG6	Glyceraldehyde-3-phosphate dehydrogenase OS=Solanum lycopersicum PE=3 SV=1
36	K4D3J1	Uncharacterized protein OS=Solanum lycopersicum PE=4 SV=1
37	K4DCW0	Uncharacterized protein OS=Solanum lycopersicum PE=3 SV=1
38	Q9FT17	Lipoxygenase OS=Solanum lycopersicum PE=2 SV=1
39	K4AXU0	Uncharacterized protein OS=Solanum lycopersicum PE=4 SV=1
40	V5YN05	Plasmamembrane intrinsic protein 15 OS=Solanum lycopersicum GN=PIP1;5 PE=2 SV=
41	K4D3E4	Fructose-bisphosphate aldolase OS=Solanum lycopersicum PE=3 SV=1
42	K4BX20	ATP synthase subunit beta OS=Solanum lycopersicum PE=3 SV=1
43	K4BNR2	ATP synthase subunit beta OS=Solanum lycopersicum PE=3 SV=1
44	K4CG46	Uncharacterized protein OS=Solanum lycopersicum PE=3 SV=1
45	K4DCQ4	Uncharacterized protein OS=Solanum lycopersicum PE=4 SV=1
46	Q4W5U7	Calnexin-like protein OS=Solanum lycopersicum GN=LeCNX61.0 PE=2 SV=1
47	P93767	ADP/ATP translocator OS=Solanum lycopersicum PE=2 SV=1
48	Q42876	Leucine aminopeptidase 2, chloroplastic OS=Solanum lycopersicum GN=LAPA2 PE=2 S

49	K4C9L4	Uncharacterized protein OS=Solanum lycopersicum PE=4 SV=1
50	K4CL75	Uncharacterized protein OS=Solanum lycopersicum PE=4 SV=1
51	K4BDY2	Uncharacterized protein OS=Solanum lycopersicum PE=4 SV=1
52	Q96575	Pectinesterase 2.2 OS=Solanum lycopersicum GN=PME2.2 PE=3 SV=1
53	Q5XP36	Glycoprotein OS=Tomato spotted wilt virus PE=4 SV=1
54	K4D4E6	Uncharacterized protein OS=Solanum lycopersicum PE=3 SV=1
55	K4BHR9	Uncharacterized protein OS=Solanum lycopersicum PE=3 SV=1
56	H1ZXA9	Heat shock protein 70 isoform 3 OS=Solanum lycopersicum GN=hsc70.3 PE=2 SV=1
57	K4D3F8	Pyruvate kinase OS=Solanum lycopersicum PE=3 SV=1
58	K4BUC5	Uncharacterized protein OS=Solanum lycopersicum PE=3 SV=1
59	K4CWR4	Adenosylhomocysteinase OS=Solanum lycopersicum PE=3 SV=1
60	K4C8R4	Glyceraldehyde-3-phosphate dehydrogenase OS=Solanum lycopersicum PE=3 SV=1
61	K7SGP9	Carboxylesterase 1 OS=Solanum lycopersicum GN=CXE1 PE=4 SV=1
62	K4CUP0	Uncharacterized protein OS=Solanum lycopersicum PE=4 SV=1
63	K4ASM0	Lipoxygenase OS=Solanum lycopersicum PE=3 SV=1
64	K4DA99	Uncharacterized protein OS=Solanum lycopersicum PE=3 SV=1
65	K4CF87	Uncharacterized protein OS=Solanum lycopersicum PE=3 SV=1
66	K4BPK3	Uncharacterized protein OS=Solanum lycopersicum PE=3 SV=1
67	Q0ZPA3	Plastid lipid associated protein CHRC OS=Solanum lycopersicum GN=ChrC PE=2 SV=1
68	K4D422	Uncharacterized protein OS=Solanum lycopersicum PE=4 SV=1
69	K4DBC4	Aconitate hydratase OS=Solanum lycopersicum PE=3 SV=1
70	P30221	17.8 kDa class I heat shock protein OS=Solanum lycopersicum PE=2 SV=1
71	Q9SYV0	17.6 kD class I small heat shock protein OS=Solanum lycopersicum GN=HSP17.6 PE=2 [illegible]
72	V5YN09	Plasmamembrane intrinsic protein 26 OS=Solanum lycopersicum GN=PIP2;6 PE=2 SV=
73	K4D9L9	Uncharacterized protein OS=Solanum lycopersicum PE=3 SV=1
74	D5L5R0	Acid invertase OS=Solanum lycopersicum var. cerasiforme GN=TAI PE=2 SV=1
75	K4B249	Uncharacterized protein OS=Solanum lycopersicum PE=3 SV=1
76	K4CFS8	Uncharacterized protein OS=Solanum lycopersicum PE=3 SV=1
77	K4BJY1	Uncharacterized protein OS=Solanum lycopersicum PE=3 SV=1
78	K4CUT2	Uncharacterized protein OS=Solanum lycopersicum PE=3 SV=1
79	K4CR90	Uncharacterized protein OS=Solanum lycopersicum PE=3 SV=1
80	K4D9L5	Uncharacterized protein OS=Solanum lycopersicum PE=3 SV=1
81	P30264	Catalase isozyme 1 OS=Solanum lycopersicum GN=CAT1 PE=2 SV=1
82	K4BUD9	Tubulin alpha chain OS=Solanum lycopersicum PE=3 SV=1
83	K4B433	Uncharacterized protein OS=Solanum lycopersicum PE=4 SV=1
84	A0A0C5CHA6	ATP synthase subunit alpha, chloroplastic OS=Solanum lycopersicum GN=atpA PE=3 S[illegible]
85	K4C1T2	Clathrin heavy chain OS=Solanum lycopersicum PE=3 SV=1
86	K4CFR0	Uncharacterized protein OS=Solanum lycopersicum PE=4 SV=1
87	K4D338	Uncharacterized protein OS=Solanum lycopersicum PE=3 SV=1
88	Q96576	Pectinesterase 3 OS=Solanum lycopersicum GN=PME3 PE=3 SV=1
89	K4CLK1	Uncharacterized protein OS=Solanum lycopersicum PE=3 SV=1
90	K4B166	Uncharacterized protein OS=Solanum lycopersicum PE=3 SV=1
91	P05117	Polygalacturonase-2 OS=Solanum lycopersicum GN=PG2 PE=1 SV=1
92	K4D2X7	Uncharacterized protein OS=Solanum lycopersicum PE=3 SV=1
93	K4CX43	Uncharacterized protein OS=Solanum lycopersicum PE=4 SV=1
94	K4DH73	Uncharacterized protein OS=Solanum lycopersicum PE=3 SV=1
95	Q6QLX4	Alcohol acyl transferase OS=Solanum lycopersicum GN=AAT1 PE=2 SV=1
96	K4B6D8	Uncharacterized protein OS=Solanum lycopersicum PE=3 SV=1
97	K4BXN9	Uncharacterized protein OS=Solanum lycopersicum PE=3 SV=1

98	K4CKY5	Uncharacterized protein OS=Solanum lycopersicum PE=3 SV=1
99	K4D6V7	Uncharacterized protein OS=Solanum lycopersicum PE=3 SV=1
100	K4DF99	Uncharacterized protein OS=Solanum lycopersicum PE=3 SV=1
101	E1Y5V2	NSs non-structural protein OS=Tomato spotted wilt virus GN=NSs PE=4 SV=1
102	K4B0D9	Uncharacterized protein OS=Solanum lycopersicum PE=4 SV=1
103	Q8S4R4	Prolycopene isomerase, chloroplastic OS=Solanum lycopersicum GN=CRTISO PE=1 SV=
104	K4ASU4	Uncharacterized protein OS=Solanum lycopersicum PE=3 SV=1
105	E0XN34	Alpha-mannosidase OS=Solanum lycopersicum PE=2 SV=1
106	K4B438	Uncharacterized protein OS=Solanum lycopersicum PE=3 SV=1
107	G5DGD2	Class I small heat shock protein 20.1 OS=Solanum lycopersicum GN=Sl20.1shsp PE=3 S
108	Q38JD4	Temperature-induced lipocalin OS=Solanum lycopersicum GN=TIL PE=2 SV=1
109	K4CW41	Uncharacterized protein OS=Solanum lycopersicum PE=4 SV=1
110	K4B3M5	V-type proton ATPase subunit a OS=Solanum lycopersicum PE=3 SV=1
111	K4CBP9	Uncharacterized protein OS=Solanum lycopersicum PE=4 SV=1
112	K4B1M2	Uncharacterized protein OS=Solanum lycopersicum PE=3 SV=1
113	K4CJ46	Isopropylmalate synthase OS=Solanum lycopersicum GN=IPMS2 PE=2 SV=1
114	K4BI68	Uncharacterized protein OS=Solanum lycopersicum PE=3 SV=1
115	K4B6W4	Uncharacterized protein OS=Solanum lycopersicum PE=3 SV=1
116	P17786	Elongation factor 1-alpha OS=Solanum lycopersicum PE=2 SV=1
117	P93207	14-3-3 protein 10 OS=Solanum lycopersicum GN=TFT10 PE=2 SV=2
118	Q6SKP4	Ribosomal protein L3 OS=Solanum lycopersicum GN=RPL3 PE=2 SV=1
119	K4BS97	Uncharacterized protein OS=Solanum lycopersicum PE=4 SV=1
120	K4BPR4	Uncharacterized protein OS=Solanum lycopersicum PE=3 SV=1
121	K4CFD4	Aconitate hydratase OS=Solanum lycopersicum PE=3 SV=1
122	K4C2D6	60S acidic ribosomal protein P0 OS=Solanum lycopersicum PE=3 SV=1
123	G5DGD4	Class I small heat shock protein OS=Solanum lycopersicum GN=Sl20.0shsp PE=3 SV=1
124	K4B6D4	Uncharacterized protein OS=Solanum lycopersicum PE=3 SV=1
125	K4BW50	Uncharacterized protein OS=Solanum lycopersicum PE=3 SV=1
126	K4CNL0	Uncharacterized protein OS=Solanum lycopersicum PE=4 SV=1
127	G8D593	Ribulose bisphosphate carboxylase large chain (Fragment) OS=Solanum lycopersicum
128	K4BP59	Glyceraldehyde-3-phosphate dehydrogenase OS=Solanum lycopersicum PE=3 SV=1
129	K4C2U0	Proteasome subunit beta type OS=Solanum lycopersicum PE=3 SV=1
130	K4C5S4	Clathrin heavy chain OS=Solanum lycopersicum PE=3 SV=1
131	P08706	Ribulose bisphosphate carboxylase small chain 1, chloroplastic OS=Solanum lycopersic
132	K4C455	Uncharacterized protein OS=Solanum lycopersicum PE=4 SV=1
133	K4D7F1	Uncharacterized protein OS=Solanum lycopersicum PE=4 SV=1
134	K4AVB7	Uncharacterized protein OS=Solanum lycopersicum PE=4 SV=1
135	K4DC02	Proteasome subunit alpha type OS=Solanum lycopersicum PE=3 SV=1
136	K4B9T4	Lactoylglutathione lyase OS=Solanum lycopersicum PE=3 SV=1
137	K4C4W6	Tubulin beta chain OS=Solanum lycopersicum PE=3 SV=1
138	K4BRG8	Uncharacterized protein OS=Solanum lycopersicum PE=3 SV=1
139	K4BVD8	Proteasome subunit alpha type OS=Solanum lycopersicum PE=3 SV=1
140	K4DB46	Uncharacterized protein OS=Solanum lycopersicum PE=4 SV=1
141	Q42887	Enolase (Fragment) OS=Solanum lycopersicum PE=4 SV=1
142	K4B3G5	Uncharacterized protein OS=Solanum lycopersicum PE=4 SV=1
143	O04936	Malic enzyme OS=Solanum lycopersicum GN=LeME2 PE=2 SV=1
144	O24024	Plastid-lipid-Associated Protein (Fragment) OS=Solanum lycopersicum GN=pap PE=4 S
145	K4CBF0	Acetyltransferase component of pyruvate dehydrogenase complex OS=Solanum lycop
146	K4BJJ6	Uncharacterized protein OS=Solanum lycopersicum PE=4 SV=1

147	K4D5U3	Glutamine synthetase OS=Solanum lycopersicum PE=3 SV=1
148	K4BJK7	40S ribosomal protein S3a OS=Solanum lycopersicum PE=3 SV=1
149	K4C745	Uncharacterized protein OS=Solanum lycopersicum PE=3 SV=1
150	O65004	Farnesyl pyrophosphate synthase OS=Solanum lycopersicum GN=FPS1 PE=2 SV=1
151	E3VWN6	1-aminocyclopropane-1-carboxylic acid oxidase OS=Solanum lycopersicum GN=ACO1
152	K4C8W9	40S ribosomal protein S3a OS=Solanum lycopersicum PE=3 SV=1
153	K4CME9	Uncharacterized protein OS=Solanum lycopersicum PE=4 SV=1
154	K4BW14	Pyrophosphate--fructose 6-phosphate 1-phosphotransferase subunit alpha OS=Solanu
155	K4CQB5	Uncharacterized protein OS=Solanum lycopersicum PE=4 SV=1
156	K4C9J5	Histone H2B OS=Solanum lycopersicum PE=3 SV=1
157	K4C3D8	40S ribosomal protein S8 OS=Solanum lycopersicum PE=3 SV=1
158	K4BA40	Proteasome subunit alpha type OS=Solanum lycopersicum PE=3 SV=1
159	K4DBV1	Uncharacterized protein OS=Solanum lycopersicum PE=3 SV=1
160	K4BY24	Uncharacterized protein OS=Solanum lycopersicum PE=4 SV=1
161	P05349	Ribulose bisphosphate carboxylase small chain 3B, chloroplastic OS=Solanum lycopers
162	Q40140	Aspartic protease OS=Solanum lycopersicum PE=2 SV=1
163	K4B4U4	Glyceraldehyde-3-phosphate dehydrogenase OS=Solanum lycopersicum PE=3 SV=1
164	K4BRF6	Uncharacterized protein OS=Solanum lycopersicum PE=4 SV=1
165	K4BL13	Uncharacterized protein OS=Solanum lycopersicum PE=3 SV=1
166	K4BVZ0	Glyceraldehyde-3-phosphate dehydrogenase OS=Solanum lycopersicum PE=3 SV=1
167	K4CE04	Uncharacterized protein OS=Solanum lycopersicum PE=3 SV=1
168	K4AYJ8	Uncharacterized protein OS=Solanum lycopersicum PE=3 SV=1
169	K4D245	Proteasome subunit alpha type OS=Solanum lycopersicum PE=3 SV=1
170	P93214	14-3-3 protein 9 OS=Solanum lycopersicum GN=TFT9 PE=2 SV=2
171	K4BPW4	Uncharacterized protein OS=Solanum lycopersicum PE=4 SV=1
172	K4BNE0	Uncharacterized protein OS=Solanum lycopersicum PE=4 SV=1
173	K4CLS9	Uncharacterized protein OS=Solanum lycopersicum PE=4 SV=1
174	K4B8V1	Uncharacterized protein OS=Solanum lycopersicum PE=3 SV=1
175	A0A0K2K0B0	Putative coat protein OS=Southern tomato virus PE=4 SV=1
176	K4D384	Uncharacterized protein OS=Solanum lycopersicum PE=4 SV=1
177	K4BWQ1	Uncharacterized protein OS=Solanum lycopersicum PE=3 SV=1
178	K4C245	Uncharacterized protein OS=Solanum lycopersicum PE=4 SV=1
179	K4D1Z3	Uncharacterized protein OS=Solanum lycopersicum PE=3 SV=1
180	K4B2T0	Uncharacterized protein OS=Solanum lycopersicum PE=4 SV=1
181	K4B1F9	Uncharacterized protein OS=Solanum lycopersicum PE=4 SV=1
182	B0JEU3	Uncharacterized protein OS=Solanum lycopersicum GN=vicilin PE=4 SV=1
183	K4CVM9	40S ribosomal protein S3a OS=Solanum lycopersicum PE=3 SV=1
184	P93212	14-3-3 protein 7 OS=Solanum lycopersicum GN=TFT7 PE=2 SV=2
185	K4DH49	Pyrophosphate--fructose 6-phosphate 1-phosphotransferase subunit alpha OS=Solanu
186	K4DHC8	Uncharacterized protein OS=Solanum lycopersicum PE=4 SV=1
187	K4D5K8	Uncharacterized protein OS=Solanum lycopersicum PE=3 SV=1
188	Q6E4P4	Carotenoid cleavage dioxygenase 1B OS=Solanum lycopersicum GN=CCD1B PE=2 SV=2
189	Q95661	Small heat shock protein, chloroplastic OS=Solanum lycopersicum GN=HSP21 PE=2 SV
190	K4B858	Uncharacterized protein OS=Solanum lycopersicum PE=4 SV=1
191	K4C9Z6	Uncharacterized protein OS=Solanum lycopersicum PE=4 SV=1
192	Q40129	Uncharacterized protein OS=Solanum lycopersicum PE=2 SV=1
193	K4CXD9	40S ribosomal protein S8 OS=Solanum lycopersicum PE=3 SV=1
194	P35057	Histone H4 OS=Solanum lycopersicum PE=3 SV=2
195	Q38JE1	Temperature-induced lipocalin OS=Solanum lycopersicum GN=TIL' PE=2 SV=1

196	K4BKP5	Uncharacterized protein OS=Solanum lycopersicum PE=3 SV=1
197	K4CAH3	40S ribosomal protein S8 OS=Solanum lycopersicum PE=3 SV=1
198	K4C261	Serine hydroxymethyltransferase OS=Solanum lycopersicum PE=3 SV=1
199	Q96489	Class II small heat shock protein Le-HSP17.6 OS=Solanum lycopersicum PE=2 SV=2
200	K4CNI4	Uncharacterized protein OS=Solanum lycopersicum PE=4 SV=1
201	K4BN29	Uncharacterized protein OS=Solanum lycopersicum PE=3 SV=1
202	K4BMN4	Uncharacterized protein OS=Solanum lycopersicum PE=3 SV=1
203	K4CGH5	Uncharacterized protein OS=Solanum lycopersicum PE=4 SV=1
204	K4B816	40S ribosomal protein S4 OS=Solanum lycopersicum PE=3 SV=1
205	K4CEY5	Uncharacterized protein OS=Solanum lycopersicum PE=3 SV=1
206	K4CEY6	Uncharacterized protein OS=Solanum lycopersicum PE=4 SV=1
207	K4BZB1	Uncharacterized protein OS=Solanum lycopersicum PE=3 SV=1
208	K4CHJ1	Uncharacterized protein OS=Solanum lycopersicum PE=4 SV=1
209	A0A097PIF5	Tomato spotted wilt virus isolate p331 putative movement protein protein (Fragment
210	K4CBD6	Xylose isomerase OS=Solanum lycopersicum PE=3 SV=1
211	K4C950	Uncharacterized protein OS=Solanum lycopersicum PE=4 SV=1
212	K4CX88	Uncharacterized protein OS=Solanum lycopersicum PE=4 SV=1
213	K4DH95	Uncharacterized protein OS=Solanum lycopersicum PE=4 SV=1
214	K4CR64	Uncharacterized protein OS=Solanum lycopersicum PE=4 SV=1
215	P37222	NADP-dependent malic enzyme, chloroplastic (Fragment) OS=Solanum lycopersicum P
216	K4B3K7	Uncharacterized protein OS=Solanum lycopersicum PE=3 SV=1
217	K4CRX6	Uncharacterized protein OS=Solanum lycopersicum PE=3 SV=1
218	K4DHE3	Uncharacterized protein OS=Solanum lycopersicum PE=4 SV=1
219	K4CCD7	Proteasome subunit beta type OS=Solanum lycopersicum PE=3 SV=1
220	K4DCU3	Uncharacterized protein OS=Solanum lycopersicum PE=4 SV=1
221	K4B110	Uncharacterized protein OS=Solanum lycopersicum PE=3 SV=1
222	K4B5N6	Uncharacterized protein OS=Solanum lycopersicum PE=4 SV=1
223	K4D6Y5	Uncharacterized protein OS=Solanum lycopersicum PE=3 SV=1
224	K4CH96	Uncharacterized protein OS=Solanum lycopersicum PE=4 SV=1
225	K4BBC4	Plastidic glucose transporter 1 OS=Solanum lycopersicum GN=pGlcT1 PE=2 SV=1
226	K4B276	Uncharacterized protein OS=Solanum lycopersicum PE=3 SV=1
227	K4AZL0	Plasmamembrane intrinsic protein 12 OS=Solanum lycopersicum GN=PIP1;2 PE=2 SV=
228	K4AYV8	Uncharacterized protein OS=Solanum lycopersicum PE=4 SV=1
229	K4D5F9	Uncharacterized protein OS=Solanum lycopersicum PE=4 SV=1
230	P54773	Photosystem II 22 kDa protein, chloroplastic OS=Solanum lycopersicum GN=PSBS PE=[illegible]
231	K4BYQ5	Uncharacterized protein OS=Solanum lycopersicum PE=4 SV=1
232	K4BVH7	Coatomer subunit alpha OS=Solanum lycopersicum PE=4 SV=1
233	K4CD10	Uncharacterized protein OS=Solanum lycopersicum PE=4 SV=1
234	K4D2W1	Uncharacterized protein OS=Solanum lycopersicum PE=3 SV=1
235	P42652	14-3-3 protein 4 OS=Solanum lycopersicum GN=TFT4 PE=2 SV=1
236	K4CWK1	Uncharacterized protein OS=Solanum lycopersicum PE=4 SV=1
237	K4CW40	Malate dehydrogenase OS=Solanum lycopersicum PE=3 SV=1
238	K4BK61	D-3-phosphoglycerate dehydrogenase OS=Solanum lycopersicum PE=3 SV=1
239	K4C891	Uncharacterized protein OS=Solanum lycopersicum PE=4 SV=1
240	K4D810	Uncharacterized protein OS=Solanum lycopersicum PE=4 SV=1
241	K4BFZ6	Uncharacterized protein OS=Solanum lycopersicum PE=4 SV=1
242	K4BJC8	Uncharacterized protein OS=Solanum lycopersicum PE=4 SV=1
243	K4CUI0	Uncharacterized protein OS=Solanum lycopersicum PE=4 SV=1
244	K4CID9	Uncharacterized protein OS=Solanum lycopersicum PE=4 SV=1

245	K4D378	Coatomer subunit beta OS=Solanum lycopersicum PE=4 SV=1
246	K4BXD4	Uncharacterized protein OS=Solanum lycopersicum PE=4 SV=1
247	K4AST1	Coatomer subunit beta OS=Solanum lycopersicum PE=4 SV=1
248	K4AYY2	Uncharacterized protein OS=Solanum lycopersicum PE=4 SV=1
249	K4B406	Uncharacterized protein OS=Solanum lycopersicum PE=4 SV=1
250	K4CUR8	Uncharacterized protein OS=Solanum lycopersicum PE=4 SV=1
251	K4CJH4	Proteasome subunit alpha type OS=Solanum lycopersicum PE=3 SV=1
252	Q5QJB4	Harpin binding protein 1 OS=Solanum lycopersicum GN=HrBP1 PE=2 SV=1
253	K4ASQ6	Uncharacterized protein OS=Solanum lycopersicum PE=4 SV=1
254	K4B832	Proteasome subunit alpha type OS=Solanum lycopersicum PE=3 SV=1
255	K4D4H7	Uncharacterized protein OS=Solanum lycopersicum PE=4 SV=1
256	K4CB65	Uncharacterized protein OS=Solanum lycopersicum PE=4 SV=1
257	K4BIL3	Uncharacterized protein OS=Solanum lycopersicum PE=4 SV=1
258	K4D3T8	Uncharacterized protein OS=Solanum lycopersicum PE=4 SV=1
259	K4CG62	Uncharacterized protein OS=Solanum lycopersicum PE=3 SV=1
260	K4CF70	Fatty acid hydroperoxide lyase, chloroplastic OS=Solanum lycopersicum GN=HPL PE=1
261	K4BJE6	Alanine--tRNA ligase OS=Solanum lycopersicum PE=3 SV=1
262	K4B171	Uncharacterized protein OS=Solanum lycopersicum PE=4 SV=1
263	K4B3H9	Uncharacterized protein OS=Solanum lycopersicum PE=4 SV=1
264	K4BNT4	Uncharacterized protein OS=Solanum lycopersicum PE=4 SV=1
265	K4BMW3	Uncharacterized protein OS=Solanum lycopersicum PE=3 SV=1
266	K4CFM5	Ketol-acid reductoisomerase OS=Solanum lycopersicum PE=3 SV=1
267	O82545	Cytosolic class II small heat shock protein HCT2 OS=Solanum lycopersicum GN=HSP17.
268	K4B407	Proteasome subunit alpha type OS=Solanum lycopersicum PE=3 SV=1
269	K4CVP8	Uncharacterized protein OS=Solanum lycopersicum PE=4 SV=1
270	K4CXJ6	Uncharacterized protein OS=Solanum lycopersicum PE=3 SV=1
271	K4D3B5	Uncharacterized protein OS=Solanum lycopersicum PE=3 SV=1
272	K4BLQ0	Uncharacterized protein OS=Solanum lycopersicum PE=3 SV=1
273	K4B838	Uncharacterized protein OS=Solanum lycopersicum PE=3 SV=1
274	K4B319	Uncharacterized protein OS=Solanum lycopersicum PE=4 SV=1
275	K4BVU7	Uncharacterized protein OS=Solanum lycopersicum PE=3 SV=1
276	K4AYP6	Uncharacterized protein OS=Solanum lycopersicum PE=4 SV=1
277	K4C3U9	Pectinesterase OS=Solanum lycopersicum PE=4 SV=1
278	K4CVK9	Uncharacterized protein OS=Solanum lycopersicum PE=4 SV=1
279	K4B5Z2	Uncharacterized protein OS=Solanum lycopersicum PE=3 SV=1
280	K4DA30	Uncharacterized protein OS=Solanum lycopersicum PE=4 SV=1
281	K4CGL2	Uncharacterized protein OS=Solanum lycopersicum PE=4 SV=1
282	K4DAS6	Uncharacterized protein OS=Solanum lycopersicum PE=3 SV=1
283	K4BT19	Uncharacterized protein OS=Solanum lycopersicum PE=3 SV=1
284	K4BV28	Uncharacterized protein OS=Solanum lycopersicum PE=3 SV=1
285	K4CHJ6	40S ribosomal protein S8 OS=Solanum lycopersicum PE=3 SV=1
286	K4CMT7	60S ribosomal protein L13 OS=Solanum lycopersicum PE=3 SV=1
287	K4B169	Uncharacterized protein OS=Solanum lycopersicum PE=3 SV=1
288	K4CB99	Uncharacterized protein OS=Solanum lycopersicum PE=3 SV=1
289	K4BB06	Proteasome subunit beta type OS=Solanum lycopersicum GN=26S beta subunit PE=3 S
290	K4C2U9	Uncharacterized protein OS=Solanum lycopersicum PE=4 SV=1
291	K4C956	Uncharacterized protein OS=Solanum lycopersicum PE=3 SV=1
292	K4B124	Uncharacterized protein OS=Solanum lycopersicum PE=4 SV=1
293	K4B3G7	Coatomer subunit gamma OS=Solanum lycopersicum PE=3 SV=1

294	K4B778	Uncharacterized protein OS=Solanum lycopersicum PE=4 SV=1
295	K4C7I1	Uncharacterized protein OS=Solanum lycopersicum PE=3 SV=1
296	O24031	Probable phospholipid hydroperoxide glutathione peroxidase OS=Solanum lycopersicu
297	K4B846	60S ribosomal protein L18a OS=Solanum lycopersicum PE=3 SV=1
298	K4AXV8	Uncharacterized protein OS=Solanum lycopersicum PE=4 SV=1
299	K4CW98	Uncharacterized protein OS=Solanum lycopersicum PE=4 SV=1
300	K4DCJ9	Uncharacterized protein OS=Solanum lycopersicum PE=4 SV=1
301	K4B0U7	Uncharacterized protein OS=Solanum lycopersicum PE=4 SV=1
302	K4DAA0	Uncharacterized protein OS=Solanum lycopersicum PE=3 SV=1
303	K4D3M1	Uncharacterized protein OS=Solanum lycopersicum PE=4 SV=1
304	K4DHD2	6,7-dimethyl-8-ribityllumazine synthase OS=Solanum lycopersicum PE=3 SV=1
305	K4C2V5	T-complex protein 1 subunit gamma OS=Solanum lycopersicum PE=3 SV=1
306	K4C7I8	60S ribosomal protein L18a OS=Solanum lycopersicum PE=3 SV=1
307	K4AYN0	Uncharacterized protein OS=Solanum lycopersicum PE=3 SV=1
308	K4BX34	Fructose-bisphosphate aldolase OS=Solanum lycopersicum PE=3 SV=1
309	K4C7C4	Uncharacterized protein OS=Solanum lycopersicum PE=4 SV=1
310	K4AYA3	Uncharacterized protein OS=Solanum lycopersicum PE=3 SV=1
311	K4BW97	Uncharacterized protein OS=Solanum lycopersicum PE=3 SV=1
312	K4AZZ5	Uncharacterized protein OS=Solanum lycopersicum PE=4 SV=1
313	K4DAV2	Uncharacterized protein OS=Solanum lycopersicum PE=4 SV=1
314	K4BKY8	Uncharacterized protein OS=Solanum lycopersicum PE=4 SV=1
315	K4BC25	Uncharacterized protein OS=Solanum lycopersicum PE=3 SV=1
316	K4CN10	Uncharacterized protein OS=Solanum lycopersicum PE=4 SV=1
317	K4CAC1	Uncharacterized protein OS=Solanum lycopersicum PE=3 SV=1
318	K4DHK7	Uncharacterized protein OS=Solanum lycopersicum PE=3 SV=1
319	K4CXX8	Proteasome subunit alpha type OS=Solanum lycopersicum PE=3 SV=1
320	K4CM58	Eukaryotic translation initiation factor 3 subunit L OS=Solanum lycopersicum PE=3 SV=
321	K4BAW0	Fructose-bisphosphate aldolase OS=Solanum lycopersicum PE=3 SV=1
322	K4AZ16	Uncharacterized protein OS=Solanum lycopersicum PE=3 SV=1
323	K4BDH9	Glucose-6-phosphate 1-dehydrogenase OS=Solanum lycopersicum PE=3 SV=1
324	K4C8T4	40S ribosomal protein SA OS=Solanum lycopersicum PE=3 SV=1
325	K4DF88	Uncharacterized protein OS=Solanum lycopersicum PE=4 SV=1
326	K4BF45	Uncharacterized protein OS=Solanum lycopersicum PE=4 SV=1
327	K4BRR1	Uncharacterized protein OS=Solanum lycopersicum PE=4 SV=1
328	K4BL38	Uncharacterized protein OS=Solanum lycopersicum PE=4 SV=1
329	A0A170RBI7	Harpin inducing protein (Fragment) OS=Solanum lycopersicum GN=Hin1 PE=2 SV=1
330	K4DFY4	Uncharacterized protein OS=Solanum lycopersicum PE=3 SV=1
331	E2GLP5	Defensin-like protein (Fragment) OS=Solanum lycopersicum var. cerasiforme PE=2 SV=
332	Q40143	Cysteine proteinase 3 OS=Solanum lycopersicum GN=CYP-3 PE=2 SV=1
333	K4BVG7	Uncharacterized protein OS=Solanum lycopersicum PE=3 SV=1
334	K4CTF6	40S ribosomal protein S8 OS=Solanum lycopersicum PE=3 SV=1
335	K4DHA3	40S ribosomal protein S6 OS=Solanum lycopersicum PE=3 SV=1
336	K4D7X4	Uncharacterized protein OS=Solanum lycopersicum PE=4 SV=1
337	G3JX11	Methionine sulfoxide reductase A OS=Solanum lycopersicum GN=MSRA PE=3 SV=1
338	Q8W5A3	Lethal leaf spot 1-like protein OS=Solanum lycopersicum PE=2 SV=1
339	K4BDC5	Uncharacterized protein OS=Solanum lycopersicum PE=4 SV=1
340	K4CI93	40S ribosomal protein S6 OS=Solanum lycopersicum PE=3 SV=1
341	G8IIJ5	Histone H2A (Fragment) OS=Solanum lycopersicum GN=HTA6 PE=2 SV=1
342	K4BG20	Uncharacterized protein OS=Solanum lycopersicum PE=3 SV=1

343	K4B111	Uncharacterized protein OS=Solanum lycopersicum PE=3 SV=1
344	K4C3E8	Uncharacterized protein OS=Solanum lycopersicum PE=3 SV=1
345	K4CB31	Transmembrane 9 superfamily member OS=Solanum lycopersicum PE=3 SV=1
346	K4BI36	Uncharacterized protein OS=Solanum lycopersicum PE=3 SV=1
347	K4CHF9	Uncharacterized protein OS=Solanum lycopersicum PE=4 SV=1
348	K4BN62	Pectinesterase OS=Solanum lycopersicum PE=4 SV=1
349	K4CG68	Uncharacterized protein OS=Solanum lycopersicum PE=4 SV=1
350	K4BAN9	Uncharacterized protein OS=Solanum lycopersicum PE=4 SV=1
351	K4D358	Uncharacterized protein OS=Solanum lycopersicum PE=4 SV=1
352	K4CSH4	Uncharacterized protein OS=Solanum lycopersicum PE=3 SV=1
353	K4CNF2	Isocitrate dehydrogenase [NAD] subunit, mitochondrial OS=Solanum lycopersicum PE[illegible]
354	K4BLN0	Uncharacterized protein OS=Solanum lycopersicum PE=4 SV=1
355	K4C3V1	Ribosomal protein L19 OS=Solanum lycopersicum PE=3 SV=1
356	P38547	GTP-binding nuclear protein Ran2 OS=Solanum lycopersicum GN=RAN2 PE=2 SV=1
357	K4C5P1	Ferritin OS=Solanum lycopersicum PE=3 SV=1
358	K4AZ93	Uncharacterized protein OS=Solanum lycopersicum PE=4 SV=1
359	B8YGR0	Cytosolic ascorbate peroxidase 2 (Fragment) OS=Solanum lycopersicum PE=2 SV=1
360	K4D831	Uncharacterized protein OS=Solanum lycopersicum PE=3 SV=1
361	K4B1M7	Uncharacterized protein OS=Solanum lycopersicum PE=4 SV=1
362	K4CR67	Histone H2A OS=Solanum lycopersicum PE=3 SV=1
363	K4CZS1	Uncharacterized protein OS=Solanum lycopersicum PE=4 SV=1
364	K4DB32	Histone H2A OS=Solanum lycopersicum PE=3 SV=1
365	K4CNR8	Uncharacterized protein OS=Solanum lycopersicum PE=4 SV=1
366	K4CHA8	Uncharacterized protein OS=Solanum lycopersicum PE=4 SV=1
367	K4B9Y6	Pyrophosphate--fructose 6-phosphate 1-phosphotransferase subunit beta OS=Solanu[illegible]
368	Q5NE21	Carbonic anhydrase OS=Solanum lycopersicum GN=ca1 PE=2 SV=1
369	K4B2Y0	Uncharacterized protein OS=Solanum lycopersicum PE=4 SV=1
370	K4D7V9	Uncharacterized protein OS=Solanum lycopersicum PE=4 SV=1
371	K4C3D3	Uncharacterized protein OS=Solanum lycopersicum PE=4 SV=1
372	K4CI69	Uncharacterized protein OS=Solanum lycopersicum PE=4 SV=1
373	K4ASC8	Uncharacterized protein OS=Solanum lycopersicum PE=3 SV=1
374	K4BMH6	Uncharacterized protein OS=Solanum lycopersicum PE=4 SV=1
375	K4CXM8	Uncharacterized protein OS=Solanum lycopersicum PE=3 SV=1
376	K4BS18	Uncharacterized protein OS=Solanum lycopersicum PE=4 SV=1
377	K4BPV3	Uncharacterized protein OS=Solanum lycopersicum PE=4 SV=1
378	K4B5E1	Uncharacterized protein OS=Solanum lycopersicum PE=4 SV=1
379	K4BF34	Uncharacterized protein OS=Solanum lycopersicum PE=4 SV=1
380	K4BLG1	Uncharacterized protein OS=Solanum lycopersicum PE=4 SV=1
381	K4DA71	Proteasome subunit beta type OS=Solanum lycopersicum PE=3 SV=1
382	K4CQB7	Uncharacterized protein OS=Solanum lycopersicum PE=3 SV=1
383	K4CM80	Uncharacterized protein OS=Solanum lycopersicum PE=4 SV=1
384	K4DBM0	Histone H2A OS=Solanum lycopersicum PE=3 SV=1
385	K4BJ57	Uncharacterized protein OS=Solanum lycopersicum PE=4 SV=1
386	K4BH28	Uncharacterized protein OS=Solanum lycopersicum PE=4 SV=1
387	K4BIB5	Uncharacterized protein OS=Solanum lycopersicum PE=4 SV=1
388	K4CXV5	Uncharacterized protein OS=Solanum lycopersicum PE=4 SV=1
389	K4CF48	Uncharacterized protein OS=Solanum lycopersicum PE=3 SV=1
390	K4B046	Uncharacterized protein OS=Solanum lycopersicum PE=3 SV=1
391	K4B1S1	Uncharacterized protein OS=Solanum lycopersicum PE=3 SV=1

392	Q66YT8	DWARF1/DIMINUTO OS=Solanum lycopersicum GN=DWF1 PE=2 SV=1
393	K4DCC6	Transmembrane 9 superfamily member OS=Solanum lycopersicum PE=3 SV=1
394	Q9SDZ6	Phospholipase D alpha (Fragment) OS=Solanum lycopersicum PE=2 SV=1
395	K4B264	Uncharacterized protein OS=Solanum lycopersicum PE=3 SV=1
396	P31542	ATP-dependent Clp protease ATP-binding subunit ClpA homolog CD4B, chloroplastic C
397	K4CXS7	Uncharacterized protein OS=Solanum lycopersicum PE=4 SV=1
398	K4CTJ3	Uncharacterized protein OS=Solanum lycopersicum PE=4 SV=1
399	B1Q3F0	Glutamate decarboxylase OS=Solanum lycopersicum GN=LeGAD1 PE=2 SV=1
400	K4BA92	Uncharacterized protein OS=Solanum lycopersicum PE=4 SV=1
401	K4AXF7	Histone H3 OS=Solanum lycopersicum PE=3 SV=1
402	K4C6M0	Ribosomal protein L15 OS=Solanum lycopersicum PE=3 SV=1
403	K4AYJ2	Uncharacterized protein OS=Solanum lycopersicum PE=4 SV=1
404	K4CMG7	Uncharacterized protein OS=Solanum lycopersicum PE=4 SV=1
405	K4BM40	T-complex protein 1 subunit delta OS=Solanum lycopersicum PE=3 SV=1
406	K4C949	60S ribosomal protein L27 OS=Solanum lycopersicum PE=3 SV=1
407	K4CJ96	6,7-dimethyl-8-ribityllumazine synthase OS=Solanum lycopersicum PE=3 SV=1
408	K4AWF0	Importin subunit alpha OS=Solanum lycopersicum PE=3 SV=1
409	K4BDP2	Phosphorus transporter OS=Solanum lycopersicum PE=2 SV=1
410	K4BT42	Uncharacterized protein OS=Solanum lycopersicum PE=4 SV=1
411	K4BYA3	Proteasome subunit beta type OS=Solanum lycopersicum PE=3 SV=1
412	Q40159	Late embryogenesis (Lea)-like protein OS=Solanum lycopersicum PE=2 SV=2
413	K4CRC9	Uncharacterized protein OS=Solanum lycopersicum PE=4 SV=1
414	K4CUX6	Elongation factor Tu OS=Solanum lycopersicum PE=3 SV=1
415	K4DBN5	Uncharacterized protein OS=Solanum lycopersicum PE=4 SV=1
416	K4CZD6	Uncharacterized protein OS=Solanum lycopersicum PE=4 SV=1
417	K4D8X9	Uncharacterized protein OS=Solanum lycopersicum PE=4 SV=1
418	K4DFU2	Uncharacterized protein OS=Solanum lycopersicum PE=3 SV=1
419	K4AXN3	Uncharacterized protein OS=Solanum lycopersicum PE=4 SV=1
420	K4B440	Uncharacterized protein OS=Solanum lycopersicum PE=4 SV=1
421	K4BDE9	Uncharacterized protein OS=Solanum lycopersicum PE=4 SV=1
422	K4CWY6	Metacaspase OS=Solanum lycopersicum GN=MCA PE=2 SV=1
423	K4BKH5	Uncharacterized protein OS=Solanum lycopersicum PE=4 SV=1
424	O82777	Subtilisin-like protease OS=Solanum lycopersicum GN=sbt3 PE=1 SV=1
425	K4DEQ8	Uncharacterized protein OS=Solanum lycopersicum PE=4 SV=1
426	P37218	Histone H1 OS=Solanum lycopersicum PE=3 SV=1
427	K4C3K6	Uncharacterized protein OS=Solanum lycopersicum PE=4 SV=1
428	K4DHI9	Uncharacterized protein OS=Solanum lycopersicum PE=4 SV=1
429	O65820	Histone H1 OS=Solanum lycopersicum GN=H1 PE=2 SV=2
430	K4CEJ5	60S ribosomal protein L27 OS=Solanum lycopersicum PE=3 SV=1
431	K4C2E1	Uncharacterized protein OS=Solanum lycopersicum PE=4 SV=1
432	K4AXF5	Uncharacterized protein OS=Solanum lycopersicum PE=4 SV=1
433	K4B546	Obg-like ATPase 1 OS=Solanum lycopersicum PE=3 SV=1
434	K4D601	Uncharacterized protein OS=Solanum lycopersicum PE=4 SV=1
435	K4B6J6	Uncharacterized protein OS=Solanum lycopersicum PE=3 SV=1
436	K4ATR9	Uncharacterized protein OS=Solanum lycopersicum PE=4 SV=1
437	K4C2U7	Uncharacterized protein OS=Solanum lycopersicum PE=3 SV=1
438	K4CN90	UMP-CMP kinase OS=Solanum lycopersicum PE=3 SV=1
439	K4BHA1	Uncharacterized protein OS=Solanum lycopersicum PE=4 SV=1
440	K4CHY4	Phosphoglycerate kinase OS=Solanum lycopersicum PE=3 SV=1

441	K4CA57	Uncharacterized protein OS=Solanum lycopersicum PE=3 SV=1
442	K4B1G9	Uncharacterized protein OS=Solanum lycopersicum PE=3 SV=1
443	K4DH61	ATP-dependent 6-phosphofructokinase OS=Solanum lycopersicum GN=PFK PE=3 SV=1
444	K4D2D7	Uncharacterized protein OS=Solanum lycopersicum PE=4 SV=1
445	K4CR73	Eukaryotic translation initiation factor 3 subunit A OS=Solanum lycopersicum PE=3 SV
446	K4DC49	Uncharacterized protein OS=Solanum lycopersicum PE=4 SV=1
447	K4C5M1	Uncharacterized protein OS=Solanum lycopersicum PE=4 SV=1
448	K4CY11	Uncharacterized protein OS=Solanum lycopersicum PE=4 SV=1
449	K4CA55	Peptidyl-prolyl cis-trans isomerase OS=Solanum lycopersicum PE=3 SV=1
450	K4C875	Uncharacterized protein OS=Solanum lycopersicum PE=4 SV=1
451	K4BVV2	Uncharacterized protein OS=Solanum lycopersicum PE=3 SV=1
452	K4BVI8	V-type proton ATPase proteolipid subunit OS=Solanum lycopersicum PE=3 SV=1
453	Q8JQG8	Nucleoprotein OS=Tomato spotted wilt virus (strain SA-05) GN=N PE=3 SV=1
454	K4BEJ6	Reticulon-like protein OS=Solanum lycopersicum PE=4 SV=1
455	K4BPK4	Uncharacterized protein OS=Solanum lycopersicum PE=3 SV=1
456	Q94K24	Ran binding protein-1 OS=Solanum lycopersicum GN=RanBP1 PE=2 SV=1
457	K4B117	Uncharacterized protein OS=Solanum lycopersicum PE=4 SV=1
458	K4AZT2	Uncharacterized protein OS=Solanum lycopersicum PE=4 SV=1
459	K4BMV8	Protein ROOT HAIR DEFECTIVE 3 homolog OS=Solanum lycopersicum PE=3 SV=1
460	K4CC33	Uncharacterized protein OS=Solanum lycopersicum PE=4 SV=1
461	K4CH35	Uncharacterized protein OS=Solanum lycopersicum PE=4 SV=1
462	K4D417	Uncharacterized protein OS=Solanum lycopersicum PE=4 SV=1
463	Q9FVH1	Transaldolase OS=Solanum lycopersicum GN=TAL PE=2 SV=1
464	K4D529	Uncharacterized protein OS=Solanum lycopersicum PE=4 SV=1
465	K4C8C0	Uncharacterized protein OS=Solanum lycopersicum PE=4 SV=1
466	K4C286	Uncharacterized protein OS=Solanum lycopersicum PE=4 SV=1
467	K4DHU1	Uncharacterized protein OS=Solanum lycopersicum PE=4 SV=1
468	Q8L7T2	Small heat shock protein OS=Solanum lycopersicum GN=vis1 PE=3 SV=1
469	K4CNT4	Uncharacterized protein OS=Solanum lycopersicum PE=4 SV=1
470	K4ASJ4	Uncharacterized protein OS=Solanum lycopersicum PE=3 SV=1
471	K4CPN0	Uncharacterized protein OS=Solanum lycopersicum PE=4 SV=1
472	K4BF68	Uncharacterized protein OS=Solanum lycopersicum PE=3 SV=1
473	K4C4X4	60S ribosomal protein L36 OS=Solanum lycopersicum PE=3 SV=1
474	K4BB90	Uncharacterized protein OS=Solanum lycopersicum PE=3 SV=1
475	K4CPR6	Uncharacterized protein OS=Solanum lycopersicum PE=4 SV=1
476	K4BPH8	Uncharacterized protein OS=Solanum lycopersicum PE=3 SV=1
477	K4C3C9	Uncharacterized protein OS=Solanum lycopersicum PE=4 SV=1
478	K4BJL4	Uncharacterized protein OS=Solanum lycopersicum PE=4 SV=1
479	K4B7Y4	Uncharacterized protein OS=Solanum lycopersicum PE=4 SV=1
480	K4DGU3	Polyadenylate-binding protein OS=Solanum lycopersicum PE=3 SV=1
481	K4CYF5	Uncharacterized protein OS=Solanum lycopersicum PE=4 SV=1
482	K4BA70	Uncharacterized protein OS=Solanum lycopersicum PE=3 SV=1
483	K4BP08	Uncharacterized protein OS=Solanum lycopersicum PE=4 SV=1
484	K4D616	Uncharacterized protein OS=Solanum lycopersicum PE=4 SV=1
485	K4CAL8	Uncharacterized protein OS=Solanum lycopersicum PE=4 SV=1
486	K4BDF6	Uncharacterized protein OS=Solanum lycopersicum PE=3 SV=1
487	K4C1V6	Phosphoinositide phospholipase C OS=Solanum lycopersicum PE=4 SV=1
488	K4C7T7	Uncharacterized protein OS=Solanum lycopersicum PE=4 SV=1
489	P47921	Nucleoside diphosphate kinase (Fragment) OS=Solanum lycopersicum PE=2 SV=1

490	K4C310	Uncharacterized protein OS=Solanum lycopersicum PE=4 SV=1
491	K4CTV6	Uncharacterized protein OS=Solanum lycopersicum PE=3 SV=1
492	K4BJG4	Uncharacterized protein OS=Solanum lycopersicum PE=3 SV=1
493	K4C4V7	Uncharacterized protein OS=Solanum lycopersicum PE=3 SV=1
494	K4BSP6	Uncharacterized protein OS=Solanum lycopersicum PE=4 SV=1
495	K4B1X9	Uncharacterized protein OS=Solanum lycopersicum PE=4 SV=1
496	K4B012	Uncharacterized protein OS=Solanum lycopersicum PE=4 SV=1
497	K4AT29	Uncharacterized protein OS=Solanum lycopersicum PE=4 SV=1
498	Q6LB28	Histone H3 OS=Solanum lycopersicum PE=2 SV=1
499	K4BJE2	Uncharacterized protein OS=Solanum lycopersicum PE=4 SV=1
500	K4BV04	Uncharacterized protein OS=Solanum lycopersicum PE=4 SV=1
501	K4BMP8	Malic enzyme OS=Solanum lycopersicum PE=3 SV=1
502	K4DA57	Uncharacterized protein OS=Solanum lycopersicum PE=3 SV=1
503	V5YN12	Tonoplast intrinsic protein 21 OS=Solanum lycopersicum GN=TIP2;1 PE=2 SV=1
504	K4CAH4	Uncharacterized protein OS=Solanum lycopersicum PE=4 SV=1
505	K4BDD3	Uncharacterized protein OS=Solanum lycopersicum PE=4 SV=1
506	K4C2J8	Uncharacterized protein OS=Solanum lycopersicum PE=4 SV=1
507	Q9XEX8	Remorin 1 OS=Solanum lycopersicum GN=rem-1 PE=2 SV=1
508	K4CF01	Uncharacterized protein OS=Solanum lycopersicum PE=4 SV=1
509	K4ATL5	Uncharacterized protein OS=Solanum lycopersicum PE=4 SV=1
510	K4B3E8	Uncharacterized protein OS=Solanum lycopersicum PE=4 SV=1
511	A6YS00	Zeta-carotene desaturase OS=Solanum lycopersicum GN=ZDS PE=2 SV=1
512	K4DF02	Uncharacterized protein OS=Solanum lycopersicum PE=3 SV=1
513	Q2PQI9	Nascent polypeptide-associated complex subunit beta OS=Solanum lycopersicum GN=
514	K4CDL3	Aspartate aminotransferase OS=Solanum lycopersicum PE=4 SV=1
515	K4D6M8	Dihydrolipoamide acetyltransferase component of pyruvate dehydrogenase complex
516	K4CX83	Glyceraldehyde-3-phosphate dehydrogenase OS=Solanum lycopersicum PE=3 SV=1
517	K4C5Y9	Uncharacterized protein OS=Solanum lycopersicum PE=4 SV=1
518	K4DB71	Uncharacterized protein OS=Solanum lycopersicum PE=4 SV=1
519	K4CAL7	Uncharacterized protein OS=Solanum lycopersicum PE=3 SV=1
520	K4D340	Uncharacterized protein OS=Solanum lycopersicum PE=4 SV=1
521	K4D2B1	Uncharacterized protein OS=Solanum lycopersicum PE=4 SV=1
522	K4BG21	Uncharacterized protein OS=Solanum lycopersicum PE=4 SV=1
523	B1Q3F8	Succinic semialdehyde dehydrogenase OS=Solanum lycopersicum GN=LeSSADH PE=2
524	K4C9A8	Uncharacterized protein OS=Solanum lycopersicum PE=4 SV=1
525	K4BJZ4	Uncharacterized protein OS=Solanum lycopersicum PE=4 SV=1
526	K4AZQ9	Reticulon-like protein OS=Solanum lycopersicum PE=4 SV=1
527	K4BMF8	Uncharacterized protein OS=Solanum lycopersicum PE=4 SV=1
528	K4CX85	Uncharacterized protein OS=Solanum lycopersicum PE=4 SV=1
529	K4DCL7	Uncharacterized protein OS=Solanum lycopersicum PE=4 SV=1
530	K4B0H4	40S ribosomal protein S24 OS=Solanum lycopersicum PE=3 SV=1
531	K4B172	Uncharacterized protein OS=Solanum lycopersicum PE=3 SV=1
532	K4D1U0	Uncharacterized protein OS=Solanum lycopersicum PE=4 SV=1
533	K4CXI9	Uncharacterized protein OS=Solanum lycopersicum PE=4 SV=1
534	K4AUV5	Uncharacterized protein OS=Solanum lycopersicum PE=4 SV=1
535	K4CWY7	Uncharacterized protein OS=Solanum lycopersicum PE=4 SV=1
536	K4BPS0	Uncharacterized protein OS=Solanum lycopersicum PE=4 SV=1
537	K4BJ07	Uncharacterized protein OS=Solanum lycopersicum PE=4 SV=1
538	Q9FR30	Ripening regulated protein DDTFR10 OS=Solanum lycopersicum GN=DDTFR10 PE=2 S\

539	K4C6V9	Uncharacterized protein OS=Solanum lycopersicum PE=4 SV=1
540	K4BKE0	Uncharacterized protein OS=Solanum lycopersicum PE=4 SV=1
541	K4D4Q9	Uncharacterized protein OS=Solanum lycopersicum PE=3 SV=1
542	K4C263	Uncharacterized protein OS=Solanum lycopersicum PE=4 SV=1
543	K4B7P2	Uncharacterized protein OS=Solanum lycopersicum PE=3 SV=1
544	K4BTH6	Peroxidase OS=Solanum lycopersicum PE=3 SV=1
545	K4CJE1	Uncharacterized protein OS=Solanum lycopersicum PE=3 SV=1
546	K4B9B8	Uncharacterized protein OS=Solanum lycopersicum PE=4 SV=1
547	K4B3I4	Response to dessication 2 OS=Solanum lycopersicum GN=Rd2 PE=2 SV=1
548	K4BV31	Uncharacterized protein OS=Solanum lycopersicum PE=3 SV=1
549	K4BN53	Uncharacterized protein OS=Solanum lycopersicum PE=3 SV=1
550	Q40133	Alcohol dehydrogenase homolog (Fragment) OS=Solanum lycopersicum GN=GAD3 PE
551	K4C2X8	Uncharacterized protein OS=Solanum lycopersicum PE=3 SV=1
552	K4D0Q5	Uncharacterized protein OS=Solanum lycopersicum PE=3 SV=1
553	K4BK69	Elongation factor Tu OS=Solanum lycopersicum PE=3 SV=1
554	K4BE37	Uncharacterized protein OS=Solanum lycopersicum PE=4 SV=1
555	K4C8Q9	Uncharacterized protein OS=Solanum lycopersicum PE=3 SV=1
556	K4C874	Uncharacterized protein OS=Solanum lycopersicum PE=4 SV=1
557	Q5NE18	Formate dehydrogenase, mitochondrial OS=Solanum lycopersicum GN=fdh PE=2 SV=1
558	K4DCH8	Oleosin OS=Solanum lycopersicum PE=3 SV=1
559	K4CS61	Uncharacterized protein OS=Solanum lycopersicum PE=3 SV=1
560	K4CB27	Uncharacterized protein OS=Solanum lycopersicum PE=4 SV=1
561	K4CZH8	Uncharacterized protein OS=Solanum lycopersicum PE=4 SV=1
562	K4D1F3	Plasmamembrane intrinsic protein 29 OS=Solanum lycopersicum GN=PIP2;9 PE=2 SV=
563	K4BMS6	Uncharacterized protein OS=Solanum lycopersicum PE=4 SV=1
564	K4BD40	Uncharacterized protein OS=Solanum lycopersicum PE=4 SV=1
565	K4CVA6	Uncharacterized protein OS=Solanum lycopersicum PE=4 SV=1
566	K4DAG8	Uncharacterized protein OS=Solanum lycopersicum PE=3 SV=1
567	K4BNV4	Uncharacterized protein OS=Solanum lycopersicum PE=3 SV=1
568	K4CHY3	Phosphoglycerate kinase OS=Solanum lycopersicum PE=3 SV=1
569	Q9FUR8	Alpha-galactosidase (Fragment) OS=Solanum lycopersicum PE=3 SV=1
570	K4CSN4	Uncharacterized protein OS=Solanum lycopersicum PE=4 SV=1
571	K4CWC4	PR10 protein OS=Solanum lycopersicum PE=2 SV=1
572	Q9AXQ6	Eukaryotic translation initiation factor 5A-1 OS=Solanum lycopersicum PE=2 SV=1
573	K4BUN4	Uncharacterized protein OS=Solanum lycopersicum PE=3 SV=1
574	K4B9E1	Uncharacterized protein OS=Solanum lycopersicum PE=4 SV=1
575	K4CET6	Uncharacterized protein OS=Solanum lycopersicum PE=4 SV=1
576	K4DHC7	Uncharacterized protein OS=Solanum lycopersicum PE=4 SV=1
577	K4BVG4	Uncharacterized protein OS=Solanum lycopersicum PE=4 SV=1
578	K4AT78	Uncharacterized protein OS=Solanum lycopersicum PE=4 SV=1
579	K4BW77	Uncharacterized protein OS=Solanum lycopersicum PE=4 SV=1
580	K4D637	Uncharacterized protein OS=Solanum lycopersicum PE=4 SV=1
581	K4B2H4	Uncharacterized protein OS=Solanum lycopersicum PE=3 SV=1
582	K4DF81	Uncharacterized protein OS=Solanum lycopersicum PE=4 SV=1
583	K4BDF9	Uncharacterized protein OS=Solanum lycopersicum PE=4 SV=1
584	K4BNR3	Uncharacterized protein OS=Solanum lycopersicum PE=4 SV=1
585	K4D276	Uncharacterized protein OS=Solanum lycopersicum PE=4 SV=1
586	K4C2B3	Uncharacterized protein OS=Solanum lycopersicum PE=4 SV=1
587	K4DA09	Uncharacterized protein OS=Solanum lycopersicum PE=4 SV=1

588	K4CLC9	Serine hydroxymethyltransferase OS=Solanum lycopersicum PE=3 SV=1
589	K4BPG2	Uncharacterized protein OS=Solanum lycopersicum PE=4 SV=1
590	Q7Y240	Thioredoxin peroxidase 1 OS=Solanum lycopersicum GN=TPx1 PE=2 SV=1
591	K4BDZ7	Uncharacterized protein OS=Solanum lycopersicum PE=4 SV=1
592	K4B0S9	Mitochondrial Rho GTPase OS=Solanum lycopersicum PE=3 SV=1
593	K4B0I7	Uncharacterized protein OS=Solanum lycopersicum PE=3 SV=1
594	K4CJ21	Malic enzyme OS=Solanum lycopersicum PE=3 SV=1
595	K4BDQ1	Uncharacterized protein OS=Solanum lycopersicum PE=4 SV=1
596	K4B4N0	Uncharacterized protein OS=Solanum lycopersicum PE=4 SV=1
597	K4BM13	Uncharacterized protein OS=Solanum lycopersicum PE=4 SV=1
598	K4DBG6	Reticulon-like protein OS=Solanum lycopersicum PE=4 SV=1
599	K4AT93	Uncharacterized protein OS=Solanum lycopersicum PE=4 SV=1
600	K4CBV3	Uncharacterized protein OS=Solanum lycopersicum PE=4 SV=1
601	K4B1S4	Uncharacterized protein OS=Solanum lycopersicum PE=3 SV=1
602	K4BLW8	Uncharacterized protein OS=Solanum lycopersicum PE=4 SV=1
603	K4D2D4	Uncharacterized protein OS=Solanum lycopersicum PE=4 SV=1
604	K4CC14	Uncharacterized protein OS=Solanum lycopersicum PE=3 SV=1
605	K4B413	Uncharacterized protein OS=Solanum lycopersicum PE=3 SV=1
606	K4CYG5	Reticulon-like protein OS=Solanum lycopersicum PE=4 SV=1
607	Q8SA58	Putative uncoupling protein OS=Solanum lycopersicum PE=2 SV=1
608	K4BQ85	Uncharacterized protein OS=Solanum lycopersicum PE=4 SV=1
609	K4BGT9	Uncharacterized protein OS=Solanum lycopersicum PE=4 SV=1
610	K4BDI6	Uncharacterized protein OS=Solanum lycopersicum PE=4 SV=1
611	K4BYC0	Uncharacterized protein OS=Solanum lycopersicum PE=3 SV=1
612	K4D4V8	Uncharacterized protein OS=Solanum lycopersicum PE=4 SV=1
613	K4BPJ4	Uncharacterized protein OS=Solanum lycopersicum PE=4 SV=1
614	K4C882	Uncharacterized protein OS=Solanum lycopersicum PE=4 SV=1
615	K4BVQ3	Uncharacterized protein OS=Solanum lycopersicum PE=4 SV=1
616	K4D533	Dihydrolipoamide acetyltransferase component of pyruvate dehydrogenase complex
617	K4D9G1	Uncharacterized protein OS=Solanum lycopersicum PE=4 SV=1
618	K4BXX0	Uncharacterized protein OS=Solanum lycopersicum PE=3 SV=1
619	K4CHN2	Uncharacterized protein OS=Solanum lycopersicum PE=4 SV=1
620	K4BVZ4	Dolichyl-diphosphooligosaccharide--protein glycosyltransferase subunit 1 OS=Solanun
621	O04359	Expansin OS=Solanum lycopersicum GN=LeEXP1 PE=2 SV=1
622	K4B0H5	Uncharacterized protein OS=Solanum lycopersicum PE=4 SV=1
623	K4BVI9	Uncharacterized protein OS=Solanum lycopersicum PE=4 SV=1
624	K4D5D2	Beta-glucosidase OS=Solanum lycopersicum PE=3 SV=1
625	K4AV20	Uncharacterized protein OS=Solanum lycopersicum PE=4 SV=1

MW [kDa]	pI	#Alt. Proteins	Scores	#Peptides	SC [%]	RMS90 [ppm]	Rank
97.1	5.6	3	3013.6 (M.score:3013.6)	42	50.2	1.35	1
28.9	9.2	97	2330.2 (M.score:2330.2)	28	58.1	0.99	2
61.5	5.5	1	1471.3 (M.score:1471.3)	28	56.1	0.90	9
28.9	9.2	98	2127.5 (M.score:2127.5)	27	62.4	0.92	4
29.0	9.0	2	2095.8 (M.score:2095.8)	27	55.0	1.06	5
28.9	9.2	150	2254.3 (M.score:2254.3)	26	52.7	0.99	3
61.1	5.8	1	1569.5 (M.score:1569.5)	26	54.1	1.00	8
65.7	4.7	2	1363.9 (M.score:1363.9)	25	46.2	0.95	12
93.3	4.9	1	1017.1 (M.score:1017.1)	25	35.8	0.97	17
80.2	5.0	1	1259.5 (M.score:1259.5)	23	30.3	0.82	13
63.0	5.7	1	1141.3 (M.score:1141.3)	23	48.7	1.07	15
61.9	5.2	4	1204.2 (M.score:1204.2)	22	45.4	0.91	14
70.2	5.2	2	1133.9 (M.score:1133.9)	22	32.9	0.79	16
23.4	9.2	2	1620.8 (M.score:1620.8)	20	56.7	0.72	6
28.8	9.3	1	1460.9 (M.score:1460.9)	19	44.6	0.97	10
60.0	6.3	3	1393.3 (M.score:1393.3)	19	41.8	0.76	11
41.0	6.0	4	880.7 (M.score:880.7)	19	55.5	0.76	20
101.1	5.8	3	802.0 (M.score:802.0)	19	28.4	0.93	29
23.0	8.8	3	1591.3 (M.score:1591.3)	18	50.7	0.74	7
347.3	8.4	21	931.0 (M.score:931.0)	18	6.0	1.09	19
80.1	5.0	1	842.0 (M.score:842.0)	18	27.8	1.03	25
80.1	5.0	1	820.8 (M.score:820.8)	18	24.6	0.77	27
170.1	4.7	1	701.1 (M.score:701.1)	18	14.3	1.03	40
347.5	8.2	3	880.5 (M.score:880.5)	17	5.7	1.17	21
53.3	7.1	1	826.4 (M.score:826.4)	17	47.9	1.11	26
52.9	6.5	30	805.4 (M.score:805.4)	17	38.6	0.73	28
38.7	7.5	3	796.6 (M.score:796.6)	17	52.0	0.82	30
110.3	5.7	3	705.1 (M.score:705.1)	17	18.5	0.94	39
110.3	6.2	3	680.9 (M.score:680.9)	17	17.6	1.05	47
68.5	5.2	5	709.1 (M.score:709.1)	16	30.7	0.92	37
55.2	7.5	1	705.4 (M.score:705.4)	16	42.7	1.17	38
36.7	7.7	4	616.2 (M.score:616.2)	16	49.6	0.89	53
96.7	5.5	4	581.7 (M.score:581.7)	16	22.4	1.13	58
41.1	5.6	2	873.3 (M.score:873.3)	15	45.2	1.30	22
36.7	6.3	3	683.5 (M.score:683.5)	15	45.9	1.36	45
89.7	5.2	4	681.9 (M.score:681.9)	15	23.3	1.29	46
110.1	5.6	2	591.8 (M.score:591.8)	15	15.5	1.15	55
97.4	5.4	3	533.1 (M.score:533.1)	15	19.3	1.04	65
105.8	6.0	1	522.3 (M.score:522.3)	15	17.1	1.22	66
30.7	8.3	7	855.2 (M.score:855.2)	14	50.7	1.48	23
38.4	8.3	1	680.1 (M.score:680.1)	14	40.8	0.99	48
59.6	5.7	2	658.9 (M.score:658.9)	14	35.0	1.72	50
59.8	5.9	1	654.9 (M.score:654.9)	14	34.7	1.64	51
109.9	5.8	2	611.7 (M.score:611.7)	14	13.5	0.95	54
44.5	7.6	2	789.1 (M.score:789.1)	13	33.2	1.26	31
61.0	4.7	2	728.5 (M.score:728.5)	13	26.0	0.94	35
42.0	9.7	5	692.2 (M.score:692.2)	13	30.3	1.30	43
59.5	8.2	5	667.2 (M.score:667.2)	13	26.9	1.14	49

89.4	5.1	1	641.8 (M.score:641.8)	13	20.9	1.25	52
92.3	5.8	3	562.0 (M.score:562.0)	13	18.6	0.83	59
56.0	6.8	1	501.1 (M.score:501.1)	13	26.6	0.91	73
60.4	8.6	2	965.6 (M.score:965.6)	12	22.9	0.78	18
127.3	5.8	80	726.9 (M.score:726.9)	12	10.1	1.39	36
41.8	5.3	8	694.4 (M.score:694.4)	12	40.3	0.57	42
41.7	5.3	1	686.8 (M.score:686.8)	12	40.3	0.56	44
71.3	5.1	11	589.0 (M.score:589.0)	12	23.7	1.37	56
57.3	6.7	3	561.2 (M.score:561.2)	12	29.0	1.04	60
73.4	6.4	4	559.2 (M.score:559.2)	12	22.2	1.71	61
53.1	5.6	4	534.5 (M.score:534.5)	12	36.1	0.93	64
36.7	7.0	2	517.6 (M.score:517.6)	12	32.3	1.03	68
37.7	5.1	5	517.6 (M.score:517.6)	12	48.4	1.28	69
58.1	8.4	1	516.3 (M.score:516.3)	12	26.7	1.35	70
101.7	6.0	5	360.0 (M.score:360.0)	12	14.8	1.07	107
30.7	8.3	2	730.8 (M.score:730.8)	11	53.0	1.53	34
55.1	5.7	2	549.0 (M.score:549.0)	11	26.6	1.49	62
41.6	5.3	9	548.6 (M.score:548.6)	11	33.2	0.75	63
35.6	5.3	1	485.3 (M.score:485.3)	11	42.3	1.27	77
39.5	5.2	1	447.7 (M.score:447.7)	11	34.0	1.06	82
107.1	6.5	1	312.4 (M.score:312.4)	11	13.1	0.71	125
17.8	5.8	4	845.5 (M.score:845.5)	10	49.4	1.04	24
17.6	5.8	2	787.5 (M.score:787.5)	10	49.4	1.23	32
30.6	8.3	1	696.9 (M.score:696.9)	10	43.2	1.47	41
71.4	5.1	2	582.1 (M.score:582.1)	10	20.0	0.85	57
70.0	5.5	7	518.8 (M.score:518.8)	10	16.5	0.88	67
41.6	5.3	1	515.6 (M.score:515.6)	10	32.6	0.59	71
41.5	9.6	1	491.6 (M.score:491.6)	10	23.8	1.30	74
52.8	6.4	1	488.8 (M.score:488.8)	10	24.5	0.71	75
54.4	7.2	1	486.1 (M.score:486.1)	10	26.6	0.75	76
71.2	5.1	4	476.2 (M.score:476.2)	10	16.9	1.24	79
77.1	5.5	1	471.7 (M.score:471.7)	10	15.8	1.28	80
56.5	6.6	3	440.6 (M.score:440.6)	10	24.2	0.93	85
49.7	4.9	5	424.7 (M.score:424.7)	10	28.4	0.86	87
55.3	8.5	2	404.8 (M.score:404.8)	10	22.2	0.97	92
55.4	5.1	3	396.6 (M.score:396.6)	10	21.9	0.86	96
192.8	5.3	2	393.1 (M.score:393.1)	10	7.8	0.85	99
90.2	5.2	1	376.0 (M.score:376.0)	10	13.7	0.95	105
84.7	6.0	3	351.5 (M.score:351.5)	10	22.0	1.36	109
60.2	8.2	1	776.8 (M.score:776.8)	9	17.1	1.01	33
50.8	6.5	4	513.8 (M.score:513.8)	9	24.5	1.69	72
39.4	5.4	1	446.5 (M.score:446.5)	9	24.8	1.26	83
50.0	6.4	1	445.1 (M.score:445.1)	9	19.7	0.50	84
30.9	8.3	3	426.4 (M.score:426.4)	9	40.4	1.39	86
21.9	5.0	1	423.8 (M.score:423.8)	9	38.8	0.44	88
44.8	5.6	6	417.8 (M.score:417.8)	9	27.4	1.16	89
49.8	7.1	3	407.0 (M.score:407.0)	9	24.2	1.37	91
36.5	5.2	1	384.7 (M.score:384.7)	9	27.7	1.12	101
89.7	4.9	1	384.4 (M.score:384.4)	9	15.2	0.75	102

26.5	9.6	2 350.8 (M.score:350.8)	9	36.6	0.44	110
74.4	5.4	2 342.2 (M.score:342.2)	9	16.0	1.64	115
93.8	5.2	2 330.5 (M.score:330.5)	9	15.7	0.88	119
52.5	8.0	210 330.2 (M.score:330.2)	9	19.5	0.55	120
36.4	6.2	2 316.8 (M.score:316.8)	9	22.2	0.54	123
67.4	6.6	1 309.5 (M.score:309.5)	9	14.6	1.41	127
65.5	5.3	6 306.3 (M.score:306.3)	9	19.9	1.35	129
116.4	6.2	2 273.3 (M.score:273.3)	9	10.1	1.30	147
53.3	5.0	2 272.8 (M.score:272.8)	9	19.0	0.93	148
17.6	5.8	1 471.5 (M.score:471.5)	8	23.4	0.91	81
21.5	6.2	1 400.9 (M.score:400.9)	8	44.9	0.97	94
57.5	6.5	1 397.7 (M.score:397.7)	8	21.2	1.18	95
92.7	5.8	2 366.9 (M.score:366.9)	8	13.3	1.43	106
118.4	6.0	7 347.4 (M.score:347.4)	8	8.0	0.63	112
21.6	7.9	1 344.1 (M.score:344.1)	8	33.3	0.67	113
66.9	5.8	2 337.9 (M.score:337.9)	8	20.8	1.62	118
73.4	5.1	4 324.3 (M.score:324.3)	8	11.4	1.45	121
122.5	5.9	2 303.4 (M.score:303.4)	8	9.4	0.75	131
49.3	9.2	3 277.1 (M.score:277.1)	8	22.1	0.62	143
28.6	4.8	1 271.8 (M.score:271.8)	8	35.3	0.90	149
44.4	10.1	2 266.4 (M.score:266.4)	8	17.2	0.52	156
50.6	7.1	2 258.4 (M.score:258.4)	8	22.9	1.29	165
28.8	4.7	9 255.5 (M.score:255.5)	8	31.4	0.45	170
108.1	7.3	1 253.9 (M.score:253.9)	8	9.9	0.62	172
34.0	5.0	2 239.9 (M.score:239.9)	8	43.0	1.42	177
17.6	5.6	2 483.5 (M.score:483.5)	7	46.8	0.70	78
36.5	5.5	4 409.6 (M.score:409.6)	7	31.8	1.47	90
20.7	6.4	7 403.8 (M.score:403.8)	7	43.6	1.26	93
31.0	6.3	2 395.7 (M.score:395.7)	7	32.3	0.90	97
21.9	7.8	1 395.2 (M.score:395.2)	7	48.5	0.71	98
42.7	8.5	1 389.5 (M.score:389.5)	7	18.5	1.19	100
29.5	6.7	2 383.1 (M.score:383.1)	7	33.7	0.49	103
192.5	5.3	1 353.4 (M.score:353.4)	7	6.0	0.55	108
20.3	7.6	1 349.3 (M.score:349.3)	7	40.3	1.32	111
47.0	5.7	2 342.3 (M.score:342.3)	7	18.2	0.65	114
47.3	5.9	1 310.7 (M.score:310.7)	7	17.9	0.53	126
123.0	4.8	1 303.2 (M.score:303.2)	7	8.7	1.17	132
27.3	6.1	1 299.4 (M.score:299.4)	7	36.6	0.90	133
38.5	6.6	2 299.1 (M.score:299.1)	7	24.3	1.77	134
50.2	4.7	10 298.6 (M.score:298.6)	7	18.3	0.74	135
63.4	6.1	1 282.4 (M.score:282.4)	7	14.2	1.54	140
32.4	5.7	1 274.7 (M.score:274.7)	7	29.1	0.88	146
40.5	6.5	1 271.6 (M.score:271.6)	7	22.2	1.03	151
35.1	6.3	3 267.6 (M.score:267.6)	7	31.0	0.99	154
23.0	5.1	3 265.5 (M.score:265.5)	7	39.3	0.98	158
64.1	5.7	4 265.4 (M.score:265.4)	7	14.7	1.18	159
15.8	4.3	1 261.2 (M.score:261.2)	7	54.8	1.31	162
59.6	8.4	1 259.1 (M.score:259.1)	7	15.6	0.80	164
34.8	5.7	1 257.8 (M.score:257.8)	7	21.8	0.84	167

38.6	5.5	6	256.0 (M.score:256.0)	7	34.7	1.18	169
29.7	9.8	1	254.4 (M.score:254.4)	7	31.4	0.81	171
47.8	5.8	2	250.2 (M.score:250.2)	7	18.2	0.63	173
39.3	5.4	3	227.4 (M.score:227.4)	7	22.2	1.06	188
35.8	5.1	2	226.8 (M.score:226.8)	7	26.0	0.94	190
29.7	9.8	1	215.4 (M.score:215.4)	7	31.4	0.72	207
51.1	9.5	3	205.2 (M.score:205.2)	7	16.9	0.98	220
67.3	6.8	1	184.8 (M.score:184.8)	7	13.6	0.59	257
44.8	10.3	1	179.1 (M.score:179.1)	7	21.2	0.85	263
15.8	10.0	12	376.5 (M.score:376.5)	6	35.7	1.07	104
25.0	10.3	1	339.9 (M.score:339.9)	6	30.0	0.82	116
27.2	5.6	1	319.9 (M.score:319.9)	6	25.8	0.43	122
54.6	6.1	2	315.2 (M.score:315.2)	6	16.5	0.70	124
47.0	5.9	2	295.2 (M.score:295.2)	6	17.3	1.07	137
20.2	6.7	3	289.9 (M.score:289.9)	6	38.3	1.35	139
55.1	5.7	1	280.5 (M.score:280.5)	6	14.6	1.28	141
43.5	8.2	1	280.2 (M.score:280.2)	6	18.1	1.27	142
29.9	5.5	1	277.1 (M.score:277.1)	6	24.6	0.79	144
35.6	9.0	1	276.5 (M.score:276.5)	6	25.3	1.23	145
50.9	6.9	2	270.2 (M.score:270.2)	6	16.2	0.61	152
26.8	9.5	1	267.4 (M.score:267.4)	6	28.5	0.77	155
58.4	5.4	1	266.3 (M.score:266.3)	6	17.7	1.23	157
27.2	6.1	2	264.4 (M.score:264.4)	6	29.3	0.97	160
29.4	4.7	4	238.0 (M.score:238.0)	6	21.5	0.43	178
17.6	8.4	2	236.3 (M.score:236.3)	6	40.1	0.89	180
40.5	5.6	5	225.3 (M.score:225.3)	6	17.9	1.40	191
40.7	6.2	1	221.5 (M.score:221.5)	6	13.7	0.39	194
63.1	5.9	2	217.0 (M.score:217.0)	6	11.4	0.66	204
42.4	5.7	7	212.4 (M.score:212.4)	6	18.3	0.92	210
25.9	9.4	3	210.5 (M.score:210.5)	6	24.4	0.97	213
23.0	10.2	2	208.4 (M.score:208.4)	6	26.9	0.43	216
108.0	5.3	1	204.5 (M.score:204.5)	6	9.7	0.41	223
65.3	5.8	2	203.7 (M.score:203.7)	6	8.6	0.84	224
69.8	5.4	1	198.5 (M.score:198.5)	6	11.3	0.47	234
82.2	4.3	1	198.1 (M.score:198.1)	6	11.3	0.79	235
66.1	8.2	1	195.3 (M.score:195.3)	6	12.5	0.66	237
29.6	9.8	1	194.7 (M.score:194.7)	6	28.5	1.05	238
28.8	5.0	2	190.2 (M.score:190.2)	6	24.6	0.57	250
67.2	7.2	1	187.0 (M.score:187.0)	6	12.5	0.60	254
62.2	8.8	1	176.4 (M.score:176.4)	6	12.8	0.89	266
28.2	4.8	2	164.6 (M.score:164.6)	6	24.9	1.23	283
61.1	5.6	3	159.1 (M.score:159.1)	6	12.7	1.27	291
26.2	7.8	2	147.4 (M.score:147.4)	6	23.4	0.84	310
31.7	8.4	2	136.2 (M.score:136.2)	6	25.0	0.58	337
88.6	8.8	1	113.3 (M.score:113.3)	6	9.5	0.99	389
25.2	9.4	1	339.4 (M.score:339.4)	5	24.4	1.24	117
25.0	10.4	1	308.0 (M.score:308.0)	5	29.0	1.57	128
11.4	11.5	3	303.6 (M.score:303.6)	5	50.5	1.07	130
21.2	6.0	1	295.3 (M.score:295.3)	5	38.4	1.21	136

21.5	6.9	1	294.7 (M.score:294.7)	5	30.3	0.73	138
24.9	10.3	1	271.7 (M.score:271.7)	5	30.0	1.78	150
52.0	6.6	3	268.8 (M.score:268.8)	5	13.0	1.05	153
17.6	6.3	1	261.4 (M.score:261.4)	5	30.4	1.68	161
39.5	6.2	1	258.2 (M.score:258.2)	5	21.0	1.65	166
57.2	5.7	1	248.5 (M.score:248.5)	5	10.4	1.01	174
64.2	5.5	1	245.0 (M.score:245.0)	5	11.1	1.32	176
25.4	5.4	9	235.0 (M.score:235.0)	5	20.9	1.28	182
29.9	10.3	5	234.4 (M.score:234.4)	5	20.8	1.25	183
90.4	5.2	1	232.1 (M.score:232.1)	5	6.9	0.46	185
29.5	8.5	1	227.5 (M.score:227.5)	5	20.3	1.21	187
47.0	8.8	5	227.0 (M.score:227.0)	5	13.2	0.89	189
82.1	5.2	1	219.7 (M.score:219.7)	5	9.0	0.98	197
22.2	8.5	191	218.7 (M.score:218.7)	5	30.1	2.11	199
44.6	5.5	1	218.3 (M.score:218.3)	5	16.1	1.80	200
21.9	9.6	2	218.3 (M.score:218.3)	5	30.9	1.37	201
17.2	6.2	13	217.2 (M.score:217.2)	5	37.0	0.89	203
81.7	8.7	3	213.8 (M.score:213.8)	5	8.2	0.86	209
23.9	7.6	7	211.1 (M.score:211.1)	5	25.9	1.01	212
63.5	6.1	6	209.1 (M.score:209.1)	5	12.4	1.71	215
44.7	8.2	2	204.5 (M.score:204.5)	5	14.3	2.02	222
17.7	5.8	2	202.8 (M.score:202.8)	5	23.4	0.79	227
22.0	9.6	2	202.1 (M.score:202.1)	5	30.9	1.44	228
25.3	5.5	1	201.8 (M.score:201.8)	5	23.3	0.74	230
24.3	5.5	12	200.4 (M.score:200.4)	5	24.0	0.82	231
47.5	4.9	3	198.7 (M.score:198.7)	5	15.6	1.68	233
25.1	8.8	1	197.9 (M.score:197.9)	5	23.4	1.24	236
21.4	5.7	2	193.3 (M.score:193.3)	5	24.2	1.05	242
51.2	7.0	1	192.7 (M.score:192.7)	5	11.4	0.89	243
57.4	9.4	1	190.4 (M.score:190.4)	5	15.6	1.96	246
24.9	9.0	1	190.2 (M.score:190.2)	5	30.2	1.20	248
30.5	8.8	1	190.2 (M.score:190.2)	5	24.9	2.38	249
22.4	5.1	6	190.1 (M.score:190.1)	5	26.6	1.37	252
36.3	9.3	1	182.2 (M.score:182.2)	5	20.0	1.67	259
29.2	8.7	1	181.6 (M.score:181.6)	5	19.2	1.01	261
25.1	7.1	1	179.2 (M.score:179.2)	5	28.3	1.47	262
137.1	6.6	2	177.4 (M.score:177.4)	5	4.9	0.57	265
28.6	6.1	1	176.4 (M.score:176.4)	5	20.2	1.10	267
64.6	5.9	2	175.4 (M.score:175.4)	5	8.8	0.61	269
29.3	4.7	2	172.0 (M.score:172.0)	5	17.3	1.58	273
108.9	5.2	1	171.9 (M.score:171.9)	5	6.3	0.74	274
35.4	5.9	1	171.6 (M.score:171.6)	5	17.5	1.31	275
63.3	7.1	2	170.9 (M.score:170.9)	5	8.8	1.20	276
64.8	5.0	1	163.3 (M.score:163.3)	5	11.4	1.25	286
89.2	7.3	2	162.9 (M.score:162.9)	5	7.0	1.11	287
59.8	8.8	1	157.8 (M.score:157.8)	5	13.4	0.88	294
29.5	5.8	3	156.9 (M.score:156.9)	5	25.5	2.93	295
80.6	6.0	2	140.8 (M.score:140.8)	5	9.9	0.71	321
30.5	9.6	1	140.4 (M.score:140.4)	5	29.1	2.34	324

101.7	5.8	1	140.2 (M.score:140.2)	5	6.8	1.68	325
65.4	4.9	2	133.2 (M.score:133.2)	5	12.8	1.41	346
105.8	5.7	1	130.2 (M.score:130.2)	5	6.5	1.70	349
36.1	7.6	1	123.2 (M.score:123.2)	5	19.9	0.86	364
48.6	8.5	1	115.3 (M.score:115.3)	5	14.0	1.32	384
15.4	10.4	2	111.2 (M.score:111.2)	5	33.8	1.10	396
26.1	4.7	1	260.1 (M.score:260.1)	4	22.4	1.42	163
30.1	6.2	1	256.9 (M.score:256.9)	4	19.2	1.34	168
17.5	5.3	1	237.9 (M.score:237.9)	4	20.8	1.28	179
26.1	4.7	1	235.3 (M.score:235.3)	4	22.4	1.41	181
25.0	8.9	2	230.7 (M.score:230.7)	4	22.5	1.12	186
51.5	7.0	1	224.9 (M.score:224.9)	4	13.2	2.01	192
19.6	6.3	1	221.6 (M.score:221.6)	4	23.5	0.97	193
22.2	6.4	2	220.5 (M.score:220.5)	4	22.6	1.01	196
20.8	9.9	2	219.3 (M.score:219.3)	4	24.9	1.18	198
53.5	6.4	2	218.0 (M.score:218.0)	4	13.2	1.70	202
110.8	6.0	1	215.7 (M.score:215.7)	4	5.1	0.56	205
18.0	6.4	1	215.6 (M.score:215.6)	4	33.1	1.47	206
17.3	5.6	2	214.4 (M.score:214.4)	4	36.0	0.66	208
16.6	6.0	1	211.7 (M.score:211.7)	4	30.6	1.29	211
60.7	7.0	1	207.3 (M.score:207.3)	4	8.7	1.19	217
63.8	6.3	2	206.8 (M.score:206.8)	4	6.2	0.79	218
17.3	6.8	2	205.5 (M.score:205.5)	4	31.0	1.23	219
27.1	7.0	3	204.7 (M.score:204.7)	4	17.7	1.26	221
38.3	5.9	1	203.2 (M.score:203.2)	4	16.5	1.07	225
21.7	5.7	2	202.8 (M.score:202.8)	4	31.0	0.68	226
16.3	10.7	1	193.7 (M.score:193.7)	4	37.3	0.77	240
80.7	5.2	7	193.5 (M.score:193.5)	4	6.4	0.98	241
16.3	10.7	1	192.2 (M.score:192.2)	4	37.3	0.67	244
62.1	8.9	1	187.7 (M.score:187.7)	4	8.1	1.26	253
23.1	5.1	1	186.9 (M.score:186.9)	4	28.2	0.48	255
23.1	7.7	3	184.2 (M.score:184.2)	4	18.8	1.41	258
62.5	9.0	2	181.8 (M.score:181.8)	4	10.8	1.54	260
22.6	6.5	1	178.3 (M.score:178.3)	4	24.5	1.40	264
37.5	9.0	3	176.4 (M.score:176.4)	4	10.9	1.06	268
37.7	5.0	1	173.9 (M.score:173.9)	4	15.7	1.13	271
25.8	7.6	1	172.5 (M.score:172.5)	4	18.8	1.21	272
24.5	4.6	1	170.6 (M.score:170.6)	4	26.0	2.83	277
30.6	10.3	2	166.3 (M.score:166.3)	4	17.5	1.73	279
42.8	5.4	1	165.7 (M.score:165.7)	4	11.3	0.60	280
25.0	10.4	1	165.4 (M.score:165.4)	4	23.1	2.04	281
23.6	11.2	2	163.3 (M.score:163.3)	4	17.5	0.70	285
22.0	6.9	4	162.5 (M.score:162.5)	4	26.4	1.01	288
30.1	8.8	2	161.8 (M.score:161.8)	4	14.7	0.75	289
24.6	5.9	1	159.1 (M.score:159.1)	4	26.5	1.03	292
60.9	6.1	1	156.7 (M.score:156.7)	4	10.1	1.88	296
17.5	10.6	3	153.6 (M.score:153.6)	4	19.1	1.07	302
20.7	10.8	1	151.5 (M.score:151.5)	4	24.6	1.63	303
98.2	5.1	1	149.8 (M.score:149.8)	4	5.0	3.08	306

29.5	8.7	1	148.7 (M.score:148.7)	4	21.7	1.76	309
60.1	6.5	1	145.7 (M.score:145.7)	4	9.0	0.77	312
18.8	6.6	2	145.7 (M.score:145.7)	4	19.5	0.35	313
21.4	10.2	3	144.2 (M.score:144.2)	4	26.4	1.28	316
168.4	6.4	4	141.2 (M.score:141.2)	4	3.1	1.16	320
44.1	5.2	1	140.5 (M.score:140.5)	4	9.8	1.51	322
23.1	6.9	3	140.5 (M.score:140.5)	4	24.6	1.81	323
77.0	6.9	1	138.8 (M.score:138.8)	4	7.9	0.90	328
17.8	8.8	4	138.5 (M.score:138.5)	4	33.7	1.15	330
23.6	10.3	2	137.1 (M.score:137.1)	4	26.2	0.86	334
24.9	7.7	1	136.5 (M.score:136.5)	4	14.7	0.77	335
60.7	5.7	1	136.2 (M.score:136.2)	4	7.9	1.54	336
21.4	10.3	1	130.5 (M.score:130.5)	4	26.4	1.26	348
73.2	8.4	5	128.8 (M.score:128.8)	4	6.8	2.29	351
43.0	8.9	1	127.1 (M.score:127.1)	4	15.4	1.04	355
64.8	4.7	1	126.8 (M.score:126.8)	4	12.9	1.03	356
26.5	9.6	1	126.7 (M.score:126.7)	4	15.9	1.56	357
16.7	10.4	3	126.6 (M.score:126.6)	4	27.2	1.03	358
109.6	5.5	1	121.8 (M.score:121.8)	4	6.1	0.47	368
19.8	5.3	2	121.2 (M.score:121.2)	4	28.0	1.49	371
40.1	5.8	3	120.9 (M.score:120.9)	4	12.5	1.80	373
59.2	6.0	1	120.1 (M.score:120.1)	4	10.0	0.98	374
37.5	4.7	1	117.6 (M.score:117.6)	4	14.2	1.32	378
46.6	5.6	2	116.9 (M.score:116.9)	4	11.1	0.70	379
23.5	10.6	3	116.8 (M.score:116.8)	4	17.0	0.48	380
25.7	5.4	2	115.2 (M.score:115.2)	4	18.3	1.40	385
60.6	5.8	1	112.5 (M.score:112.5)	4	8.1	0.88	392
42.4	8.1	3	111.9 (M.score:111.9)	4	10.7	0.36	395
59.2	6.0	1	110.1 (M.score:110.1)	4	9.7	1.45	398
58.5	6.4	1	105.5 (M.score:105.5)	4	7.6	1.64	404
32.2	4.9	2	105.3 (M.score:105.3)	4	16.2	1.45	405
104.5	4.9	2	97.3 (M.score:97.3)	4	4.3	0.99	433
56.4	6.5	1	97.0 (M.score:97.0)	4	8.0	0.73	435
12.5	6.8	2	95.9 (M.score:95.9)	4	30.6	0.86	443
16.1	10.2	1	94.1 (M.score:94.1)	4	24.5	2.07	451
26.1	9.3	1	85.8 (M.score:85.8)	4	16.4	2.00	476
37.3	5.7	2	85.7 (M.score:85.7)	4	14.4	0.70	478
5.3	8.8	3	247.0 (M.score:247.0)	3	50.0	1.08	175
38.9	8.6	1	233.2 (M.score:233.2)	3	8.4	0.54	184
95.7	6.1	1	220.8 (M.score:220.8)	3	5.4	1.11	195
25.1	10.3	1	209.8 (M.score:209.8)	3	18.9	2.30	214
28.3	10.6	1	201.8 (M.score:201.8)	3	15.7	2.03	229
18.4	5.3	1	199.4 (M.score:199.4)	3	19.2	1.16	232
21.1	6.1	3	193.9 (M.score:193.9)	3	23.9	0.84	239
60.6	6.5	2	191.7 (M.score:191.7)	3	8.0	0.57	245
62.2	6.1	2	190.1 (M.score:190.1)	3	7.7	1.15	251
28.3	10.7	2	174.4 (M.score:174.4)	3	15.7	1.92	270
15.3	10.5	4	168.7 (M.score:168.7)	3	41.8	1.73	278
16.7	10.8	3	165.0 (M.score:165.0)	3	23.3	0.37	282

18.9	4.6	3	164.3 (M.score:164.3)	3	22.6	1.35	284
34.4	9.4	2	160.5 (M.score:160.5)	3	16.3	1.01	290
73.7	6.5	1	156.2 (M.score:156.2)	3	7.0	1.23	297
21.6	5.8	1	156.1 (M.score:156.1)	3	15.8	0.78	298
51.1	9.1	1	154.7 (M.score:154.7)	3	9.4	1.85	299
64.0	8.9	2	153.9 (M.score:153.9)	3	6.3	1.03	300
24.1	5.7	2	153.9 (M.score:153.9)	3	16.1	0.64	301
42.7	4.4	1	150.9 (M.score:150.9)	3	9.4	0.87	304
49.4	5.7	1	150.7 (M.score:150.7)	3	7.8	1.10	305
34.5	9.5	1	149.8 (M.score:149.8)	3	15.0	1.47	307
40.6	6.8	2	148.9 (M.score:148.9)	3	9.5	0.94	308
58.6	6.8	1	146.3 (M.score:146.3)	3	8.6	0.79	311
24.7	11.5	2	145.1 (M.score:145.1)	3	12.2	1.18	314
25.1	6.4	2	144.4 (M.score:144.4)	3	14.5	0.89	315
28.2	5.8	2	143.6 (M.score:143.6)	3	18.7	1.34	317
43.0	5.6	1	139.4 (M.score:139.4)	3	11.6	1.26	326
25.7	6.1	5	138.5 (M.score:138.5)	3	19.7	1.94	329
23.6	9.5	2	137.8 (M.score:137.8)	3	14.2	0.81	331
134.6	5.7	1	137.5 (M.score:137.5)	3	3.2	1.69	332
15.4	10.5	3	137.2 (M.score:137.2)	3	42.1	1.28	333
23.9	8.3	1	135.9 (M.score:135.9)	3	14.4	0.48	338
14.9	10.8	2	134.9 (M.score:134.9)	3	43.3	1.69	340
66.1	8.4	1	134.7 (M.score:134.7)	3	5.8	1.95	341
43.2	9.8	2	134.3 (M.score:134.3)	3	11.9	1.31	342
61.7	6.0	2	133.2 (M.score:133.2)	3	9.7	0.80	345
29.5	5.6	1	131.2 (M.score:131.2)	3	13.4	0.67	347
29.9	10.5	1	128.6 (M.score:128.6)	3	18.7	1.43	352
82.3	6.8	1	127.7 (M.score:127.7)	3	4.2	1.32	354
26.0	10.4	4	126.4 (M.score:126.4)	3	13.5	0.80	359
33.3	9.2	1	124.5 (M.score:124.5)	3	12.7	1.44	360
31.7	9.5	2	124.2 (M.score:124.2)	3	9.8	0.74	361
103.5	4.8	1	123.7 (M.score:123.7)	3	3.5	0.79	362
32.7	6.3	1	123.4 (M.score:123.4)	3	14.9	1.93	363
34.0	6.6	1	122.3 (M.score:122.3)	3	14.7	2.06	366
14.9	9.0	1	122.2 (M.score:122.2)	3	38.9	1.08	367
28.9	5.6	1	121.1 (M.score:121.1)	3	16.4	0.79	372
44.3	5.2	1	119.4 (M.score:119.4)	3	8.4	1.64	375
21.6	9.0	1	119.4 (M.score:119.4)	3	18.5	1.08	376
27.6	7.0	1	117.6 (M.score:117.6)	3	19.6	1.33	377
27.6	5.6	2	116.1 (M.score:116.1)	3	11.6	1.58	381
55.5	6.1	1	115.9 (M.score:115.9)	3	7.8	1.38	382
14.5	10.2	2	115.1 (M.score:115.1)	3	35.6	1.83	386
13.9	10.5	3	114.9 (M.score:114.9)	3	28.3	1.10	387
32.6	7.5	1	114.8 (M.score:114.8)	3	10.5	0.81	388
33.6	5.4	1	113.2 (M.score:113.2)	3	14.2	1.93	391
36.0	7.2	1	111.1 (M.score:111.1)	3	14.3	2.19	397
47.0	5.6	1	108.2 (M.score:108.2)	3	9.7	0.32	402
13.1	9.7	2	104.6 (M.score:104.6)	3	26.5	1.54	406
74.9	5.2	1	102.5 (M.score:102.5)	3	5.4	1.81	411

66.2	7.6	2	101.7 (M.score:101.7)	3	5.6	1.38	414
74.2	6.1	4	101.5 (M.score:101.5)	3	5.3	0.98	415
92.1	5.4	2	101.4 (M.score:101.4)	3	5.6	1.33	416
76.0	5.8	1	101.2 (M.score:101.2)	3	6.2	2.93	418
102.2	5.9	4	101.0 (M.score:101.0)	3	4.6	1.22	419
66.3	6.3	4	99.3 (M.score:99.3)	3	8.1	0.60	422
34.5	9.1	1	98.0 (M.score:98.0)	3	9.4	1.61	428
56.7	5.9	2	97.8 (M.score:97.8)	3	5.6	0.58	430
113.7	8.6	1	97.6 (M.score:97.6)	3	4.1	0.91	431
15.2	11.3	4	97.0 (M.score:97.0)	3	16.9	0.79	434
24.1	11.4	3	96.6 (M.score:96.6)	3	13.7	0.93	437
121.5	5.2	1	96.6 (M.score:96.6)	3	3.7	2.16	438
13.6	8.5	1	96.1 (M.score:96.1)	3	31.3	1.00	441
58.0	6.7	1	95.9 (M.score:95.9)	3	6.3	1.53	442
15.6	10.5	1	95.3 (M.score:95.3)	3	22.2	0.78	444
25.1	7.7	1	95.2 (M.score:95.2)	3	10.3	0.46	445
58.7	5.1	1	95.0 (M.score:95.0)	3	6.4	0.85	447
38.4	9.2	3	94.6 (M.score:94.6)	3	12.6	1.34	448
23.3	5.4	1	92.9 (M.score:92.9)	3	16.7	1.17	452
26.8	6.7	2	91.3 (M.score:91.3)	3	10.8	1.06	456
19.1	4.6	2	91.3 (M.score:91.3)	3	26.4	1.09	457
43.4	5.0	1	90.9 (M.score:90.9)	3	7.1	1.41	458
49.0	6.5	2	90.6 (M.score:90.6)	3	8.2	1.17	459
78.6	6.2	2	90.3 (M.score:90.3)	3	3.9	2.05	460
45.8	6.7	1	88.7 (M.score:88.7)	3	7.5	1.85	464
39.9	8.6	1	87.7 (M.score:87.7)	3	10.4	0.80	466
26.1	6.3	5	86.7 (M.score:86.7)	3	12.7	0.89	471
25.2	5.2	1	86.6 (M.score:86.6)	3	15.3	3.17	472
99.2	4.8	1	86.1 (M.score:86.1)	3	3.8	1.19	474
23.3	7.7	1	86.1 (M.score:86.1)	3	19.0	1.65	475
45.5	4.8	2	85.3 (M.score:85.3)	3	10.6	1.05	479
31.3	5.7	3	83.2 (M.score:83.2)	3	9.5	2.69	487
82.2	8.2	8	82.3 (M.score:82.3)	3	4.5	0.64	491
16.0	10.1	1	81.7 (M.score:81.7)	3	16.8	0.82	492
29.9	10.7	1	80.1 (M.score:80.1)	3	7.3	0.34	496
15.4	10.3	2	78.1 (M.score:78.1)	3	21.6	0.59	506
36.9	7.6	1	75.5 (M.score:75.5)	3	8.8	1.96	516
28.8	10.5	1	75.0 (M.score:75.0)	3	11.1	2.44	519
15.6	10.3	1	73.0 (M.score:73.0)	3	22.2	1.05	525
107.5	6.8	2	71.3 (M.score:71.3)	3	2.7	0.78	534
60.4	9.0	1	71.0 (M.score:71.0)	3	6.3	2.41	535
44.4	6.6	1	70.7 (M.score:70.7)	3	10.9	1.08	537
51.8	5.8	2	70.1 (M.score:70.1)	3	6.7	0.38	541
58.8	6.3	1	69.9 (M.score:69.9)	3	5.4	1.26	543
29.4	7.8	1	67.8 (M.score:67.8)	3	11.2	2.48	547
50.9	6.1	2	67.2 (M.score:67.2)	3	9.2	1.25	552
24.4	7.6	1	66.0 (M.score:66.0)	3	16.2	1.95	556
177.0	6.1	1	65.8 (M.score:65.8)	3	3.2	2.03	559
50.4	7.7	1	63.4 (M.score:63.4)	3	6.6	2.00	568

33.1	5.9	1 63.1 (M.score:63.1)	3	11.0	0.96	569
84.1	6.9	1 61.9 (M.score:61.9)	3	4.3	0.96	573
53.8	6.1	5 59.2 (M.score:59.2)	3	6.2	1.31	583
18.7	10.6	2 58.3 (M.score:58.3)	3	15.9	1.23	587
118.0	9.3	2 57.6 (M.score:57.6)	3	2.9	0.96	592
75.1	5.3	2 57.6 (M.score:57.6)	3	4.9	1.74	593
32.5	5.0	1 53.9 (M.score:53.9)	3	10.4	0.70	612
59.8	6.0	1 53.8 (M.score:53.8)	3	1.7	1.31	613
22.2	9.2	2 52.7 (M.score:52.7)	3	13.0	0.72	615
59.4	5.2	1 50.3 (M.score:50.3)	3	6.4	0.68	630
75.9	6.1	2 47.8 (M.score:47.8)	3	7.1	0.85	643
16.6	7.8	3 190.3 (M.score:190.3)	2	28.5	0.88	247
28.7	8.9	1 185.8 (M.score:185.8)	2	3.1	1.75	256
28.7	6.7	1 158.1 (M.score:158.1)	2	13.3	0.92	293
27.5	5.5	1 142.4 (M.score:142.4)	2	10.4	0.76	318
25.0	4.9	1 142.3 (M.score:142.3)	2	20.5	0.77	319
14.0	9.5	3 139.0 (M.score:139.0)	2	18.4	0.50	327
18.0	9.0	1 135.1 (M.score:135.1)	2	17.6	2.46	339
90.7	5.5	2 133.9 (M.score:133.9)	2	3.6	1.25	344
44.3	5.8	1 129.5 (M.score:129.5)	2	7.0	2.07	350
34.3	8.3	1 128.1 (M.score:128.1)	2	5.7	0.66	353
40.2	5.6	1 122.3 (M.score:122.3)	2	7.2	1.04	365
47.6	5.6	2 121.8 (M.score:121.8)	2	5.3	0.81	369
15.2	5.2	1 121.6 (M.score:121.6)	2	27.5	1.67	370
45.3	5.5	1 115.5 (M.score:115.5)	2	6.9	2.20	383
25.6	10.2	2 113.2 (M.score:113.2)	2	9.6	0.65	390
18.8	8.3	2 112.3 (M.score:112.3)	2	15.2	0.88	393
25.7	9.3	2 112.1 (M.score:112.1)	2	10.0	1.24	394
45.6	4.9	1 109.6 (M.score:109.6)	2	6.4	0.71	399
67.3	6.5	1 109.5 (M.score:109.5)	2	4.1	0.97	400
42.7	9.1	1 107.2 (M.score:107.2)	2	8.9	2.39	403
24.7	4.5	1 104.5 (M.score:104.5)	2	14.5	0.89	407
15.7	11.5	3 104.1 (M.score:104.1)	2	13.0	0.13	408
58.9	6.0	1 103.7 (M.score:103.7)	2	4.1	1.46	409
30.7	5.2	1 102.6 (M.score:102.6)	2	7.1	0.76	410
14.3	10.8	3 102.3 (M.score:102.3)	2	16.3	0.98	412
14.6	10.9	2 102.2 (M.score:102.2)	2	18.9	2.21	413
15.0	8.5	1 101.2 (M.score:101.2)	2	17.8	0.45	417
54.0	5.3	1 99.9 (M.score:99.9)	2	5.8	0.74	420
72.2	6.7	1 99.4 (M.score:99.4)	2	4.4	1.25	421
18.1	6.7	1 99.0 (M.score:99.0)	2	17.2	0.99	423
17.3	10.4	1 98.7 (M.score:98.7)	2	19.2	1.46	424
38.0	8.5	1 98.6 (M.score:98.6)	2	6.2	0.25	425
25.4	10.1	1 98.2 (M.score:98.2)	2	9.6	0.80	426
32.8	9.0	2 98.0 (M.score:98.0)	2	7.8	0.63	427
15.6	7.6	1 97.9 (M.score:97.9)	2	19.9	1.40	429
66.2	5.9	2 97.4 (M.score:97.4)	2	4.6	1.37	432
15.9	9.2	1 96.9 (M.score:96.9)	2	16.3	0.71	436
15.7	7.0	4 96.4 (M.score:96.4)	2	17.4	1.53	439

11.3	7.7	2	96.4 (M.score:96.4)	2	19.4	1.32	440
14.7	5.2	3	95.1 (M.score:95.1)	2	26.3	1.29	446
45.8	6.2	1	94.5 (M.score:94.5)	2	5.6	1.61	449
25.8	4.4	1	94.2 (M.score:94.2)	2	12.5	1.15	450
20.9	5.8	1	92.6 (M.score:92.6)	2	15.4	1.03	453
185.7	5.7	1	92.2 (M.score:92.2)	2	1.2	0.75	455
30.5	9.3	1	90.3 (M.score:90.3)	2	12.0	0.23	461
64.7	5.4	1	89.7 (M.score:89.7)	2	4.0	0.68	462
15.4	11.2	2	89.5 (M.score:89.5)	2	10.3	0.90	463
17.5	9.5	1	87.5 (M.score:87.5)	2	12.9	0.94	467
34.7	6.2	1	87.4 (M.score:87.4)	2	8.0	1.22	468
70.9	7.2	1	87.3 (M.score:87.3)	2	3.1	1.19	469
56.9	5.7	1	87.0 (M.score:87.0)	2	6.3	0.90	470
25.1	5.7	7	86.5 (M.score:86.5)	2	18.1	2.08	473
63.5	5.2	2	85.8 (M.score:85.8)	2	3.7	0.19	477
35.7	6.0	1	85.1 (M.score:85.1)	2	5.9	0.27	480
48.9	5.4	4	84.7 (M.score:84.7)	2	5.7	0.41	481
21.8	5.6	1	84.6 (M.score:84.6)	2	16.2	0.69	482
42.4	5.3	1	84.2 (M.score:84.2)	2	12.0	1.31	483
17.7	4.8	1	84.1 (M.score:84.1)	2	31.7	0.65	484
83.1	5.6	1	83.6 (M.score:83.6)	2	3.4	1.27	486
64.5	8.4	4	82.4 (M.score:82.4)	2	4.3	1.39	490
17.6	10.5	2	81.2 (M.score:81.2)	2	10.7	0.34	493
17.4	6.8	3	80.9 (M.score:80.9)	2	18.5	1.46	494
39.2	8.2	2	80.2 (M.score:80.2)	2	7.0	1.31	495
48.8	8.4	1	79.9 (M.score:79.9)	2	7.3	2.99	497
44.5	8.6	1	79.8 (M.score:79.8)	2	3.3	1.06	498
86.7	4.5	2	79.6 (M.score:79.6)	2	3.6	2.08	499
28.2	10.8	1	79.5 (M.score:79.5)	2	8.8	1.57	500
49.4	5.9	1	78.9 (M.score:78.9)	2	5.4	0.33	502
40.7	4.9	1	78.8 (M.score:78.8)	2	5.4	1.06	503
22.3	9.3	1	78.6 (M.score:78.6)	2	18.3	0.36	504
29.0	8.8	1	78.2 (M.score:78.2)	2	8.0	2.02	505
55.8	7.5	1	77.6 (M.score:77.6)	2	3.4	0.27	507
16.8	6.3	2	77.2 (M.score:77.2)	2	12.2	1.46	508
47.4	5.4	1	76.7 (M.score:76.7)	2	5.6	0.74	509
29.7	7.8	1	76.4 (M.score:76.4)	2	6.8	2.15	511
32.7	5.2	1	75.8 (M.score:75.8)	2	7.5	0.60	512
98.1	5.1	1	75.8 (M.score:75.8)	2	2.4	0.51	513
25.6	6.9	1	75.7 (M.score:75.7)	2	8.7	1.73	514
15.4	10.6	3	75.5 (M.score:75.5)	2	18.0	0.25	515
47.6	4.5	1	75.2 (M.score:75.2)	2	4.3	0.53	517
69.2	5.3	2	75.2 (M.score:75.2)	2	3.7	2.46	518
28.2	10.8	3	74.0 (M.score:74.0)	2	6.9	0.50	521
10.7	6.4	3	73.2 (M.score:73.2)	2	34.0	0.70	523
24.3	5.1	7	73.1 (M.score:73.1)	2	8.8	0.55	524
24.6	8.4	4	72.3 (M.score:72.3)	2	8.6	0.55	527
66.2	5.9	1	72.1 (M.score:72.1)	2	5.6	1.24	528
22.2	4.7	2	72.0 (M.score:72.0)	2	13.7	1.25	529

25.3	6.3	1 72.0 (M.score:72.0)	2	11.6	1.62	530
44.8	10.0	1 71.9 (M.score:71.9)	2	10.2	1.20	531
46.6	6.1	5 71.6 (M.score:71.6)	2	6.4	1.51	532
86.2	5.4	2 70.6 (M.score:70.6)	2	4.2	1.57	538
38.9	7.9	1 70.3 (M.score:70.3)	2	9.3	1.18	539
38.1	6.1	2 70.0 (M.score:70.0)	2	6.6	1.42	542
50.0	7.1	1 68.5 (M.score:68.5)	2	5.1	1.85	544
34.6	6.8	1 68.4 (M.score:68.4)	2	5.2	1.01	545
19.4	6.0	1 67.9 (M.score:67.9)	2	13.6	1.11	546
43.2	5.5	3 67.7 (M.score:67.7)	2	6.2	0.99	549
16.1	8.4	1 67.2 (M.score:67.2)	2	16.0	1.59	551
26.2	5.6	2 66.8 (M.score:66.8)	2	11.6	1.38	553
46.1	7.9	1 65.8 (M.score:65.8)	2	4.4	0.51	557
59.9	9.3	1 65.8 (M.score:65.8)	2	3.7	0.74	558
51.8	6.1	2 65.0 (M.score:65.0)	2	5.7	2.04	561
83.8	7.9	1 64.9 (M.score:64.9)	2	3.0	1.18	562
20.0	10.1	3 64.8 (M.score:64.8)	2	10.9	2.30	563
35.9	6.7	5 64.4 (M.score:64.4)	2	7.7	1.96	565
42.1	6.9	1 63.6 (M.score:63.6)	2	5.5	0.53	566
18.2	9.7	1 63.5 (M.score:63.5)	2	12.2	0.62	567
60.8	6.6	9 63.1 (M.score:63.1)	2	3.4	0.92	570
28.5	10.1	1 62.5 (M.score:62.5)	2	7.3	0.79	571
32.9	9.4	3 62.0 (M.score:62.0)	2	5.5	1.66	572
30.3	8.4	2 61.5 (M.score:61.5)	2	10.6	1.26	574
48.7	5.3	1 61.2 (M.score:61.2)	2	5.6	1.39	575
29.3	8.9	1 60.2 (M.score:60.2)	2	7.6	2.03	577
20.8	10.8	1 59.9 (M.score:59.9)	2	11.2	1.26	580
24.3	4.9	3 59.5 (M.score:59.5)	2	9.2	0.82	582
41.3	5.3	1 58.6 (M.score:58.6)	2	5.5	0.80	584
42.3	5.8	1 58.2 (M.score:58.2)	2	4.5	1.39	588
41.8	4.9	3 57.6 (M.score:57.6)	2	8.4	0.90	591
111.5	5.7	1 56.9 (M.score:56.9)	2	2.7	1.21	595
17.4	5.4	2 56.3 (M.score:56.3)	2	16.9	0.89	598
17.3	5.7	7 56.3 (M.score:56.3)	2	11.3	0.22	599
28.9	6.9	2 56.0 (M.score:56.0)	2	7.5	1.65	601
65.9	8.2	1 55.9 (M.score:55.9)	2	3.1	1.92	602
79.8	8.3	1 55.6 (M.score:55.6)	2	3.4	1.11	603
19.8	9.8	2 55.3 (M.score:55.3)	2	13.9	1.62	604
13.6	5.9	2 55.2 (M.score:55.2)	2	14.6	1.62	606
37.4	7.6	1 54.9 (M.score:54.9)	2	6.5	1.27	607
66.4	4.8	1 53.7 (M.score:53.7)	2	4.5	1.62	614
15.3	3.5	1 52.6 (M.score:52.6)	2	16.9	1.65	617
90.7	6.3	4 52.5 (M.score:52.5)	2	3.0	0.48	618
35.7	6.5	2 52.3 (M.score:52.3)	2	4.6	1.02	619
65.5	6.6	1 52.1 (M.score:52.1)	2	3.7	0.67	620
101.6	6.0	1 51.9 (M.score:51.9)	2	2.5	1.48	622
53.8	8.6	1 51.4 (M.score:51.4)	2	3.5	2.28	623
36.2	5.8	4 51.3 (M.score:51.3)	2	6.9	1.10	625
12.3	9.3	1 50.8 (M.score:50.8)	2	32.5	0.24	628

57.0	7.2	2	50.2 (M.score:50.2)	2	4.4	1.23	631
62.1	5.0	1	49.7 (M.score:49.7)	2	3.5	1.19	633
17.4	5.2	1	49.5 (M.score:49.5)	2	18.5	0.93	634
55.2	7.0	1	49.1 (M.score:49.1)	2	5.7	1.09	637
71.5	5.3	2	47.9 (M.score:47.9)	2	3.9	1.54	640
26.1	4.5	1	47.9 (M.score:47.9)	2	12.4	1.22	641
69.9	5.8	1	47.5 (M.score:47.5)	2	4.0	2.87	645
26.9	5.7	1	47.3 (M.score:47.3)	2	8.0	4.76	647
65.7	5.3	1	45.9 (M.score:45.9)	2	3.6	0.44	658
144.1	6.0	1	45.7 (M.score:45.7)	2	2.2	0.23	660
25.9	8.9	1	45.5 (M.score:45.5)	2	9.0	1.57	664
41.8	4.7	1	45.3 (M.score:45.3)	2	7.4	1.47	666
64.7	7.5	2	44.4 (M.score:44.4)	2	3.8	0.83	670
57.8	5.5	2	44.0 (M.score:44.0)	2	4.9	0.84	672
70.1	5.8	1	42.8 (M.score:42.8)	2	3.1	3.79	679
39.4	5.9	1	42.7 (M.score:42.7)	2	9.7	1.45	680
91.3	6.8	1	42.6 (M.score:42.6)	2	2.8	1.01	681
31.6	7.1	4	41.9 (M.score:41.9)	2	7.7	3.25	686
29.4	7.7	1	41.6 (M.score:41.6)	2	5.3	0.45	688
32.4	9.4	1	40.2 (M.score:40.2)	2	5.2	0.63	694
103.9	5.6	2	39.7 (M.score:39.7)	2	3.3	0.25	698
36.4	9.0	1	39.6 (M.score:39.6)	2	9.2	2.12	699
29.5	5.7	1	39.6 (M.score:39.6)	2	10.8	1.02	700
59.1	5.6	1	39.3 (M.score:39.3)	2	3.7	0.30	703
31.9	5.2	1	39.0 (M.score:39.0)	2	7.2	0.68	705
38.5	6.4	8	37.6 (M.score:37.6)	2	6.4	1.13	715
22.1	9.8	2	36.4 (M.score:36.4)	2	17.3	1.76	721
43.5	9.1	1	35.5 (M.score:35.5)	2	6.0	3.32	727
68.6	5.9	1	34.9 (M.score:34.9)	2	3.1	0.29	734
43.8	8.8	1	34.0 (M.score:34.0)	2	7.5	1.24	741
59.5	6.2	1	33.5 (M.score:33.5)	2	5.4	2.17	743
43.6	5.6	1	33.3 (M.score:33.3)	2	6.8	0.86	746
69.0	7.3	1	31.0 (M.score:31.0)	2	2.8	1.25	761
28.7	9.5	6	29.3 (M.score:29.3)	2	6.1	0.04	775
20.2	5.3	1	29.2 (M.score:29.2)	2	13.4	3.61	780
227.7	5.4	1	28.8 (M.score:28.8)	2	0.8	0.43	782
57.7	5.1	1	28.4 (M.score:28.4)	2	3.4	0.77	783
37.0	9.3	1	28.0 (M.score:28.0)	2	4.1	5.73	787
